# Genome-Wide Analysis of *Triticum aestivum* Root Meristem Growth Factor (RGF) Gene Family Highlights TaRGF5 as a Putative Component of Root-Associated Signaling

**DOI:** 10.3390/ijms27177616

**Published:** 2026-08-25

**Authors:** Hala B. Khalil, Haidar A. Alsahoud, Abdulrahman Darwish Mostafa, Fatimah A. Alhassan, Norah Al-helal, Shinya Ikeno

**Affiliations:** 1 Biological Sciences Department, College of Science, King Faisal University, Al-Ahsa 31982, Saudi Arabia; 2 Genetics Department, Faculty of Agriculture, Ain Shams University, 68 Hadayek Shoubra, Cairo 11241, Egypt; 3 Al-Anjal Private Schools, Al-Ahsa 36361, Saudi Arabia; 4 Department of Biological Functions and Engineering, Graduate School of Life Science and System Engineering, Kyushu Institute of Technology, 2-4 Hibikino, Wakamatsu Ward, Kitakyushu 808-0196, Japan

**Keywords:** *Triticum aestivum*, root meristem growth factors, drought stress, root development, RNA-seq, molecular docking, molecular dynamics simulation, root system architecture

## Abstract

Wheat (*Triticum aestivum*), a key global crop, faces rising drought stress that limits root growth and water uptake. Root meristem growth factors (RGFs) are small peptides that regulate root stem cell maintenance, meristem activity, and lateral root formation in model plants, yet the RGF gene family remains unexplored in wheat. Here, we performed a comprehensive genome-wide analysis of the TaRGF gene family, identifying 15 genes distributed across the A, B, and D subgenomes and classified into five homeologous groups (*TaRGF1*–*TaRGF5*), predominantly located on chromosomes 2 and 6. All TaRGFs contained a characteristic RGF motif, with dibasic cleavage sites and Asp–Tyr motifs indicating conserved maturation mechanisms. Based on the phylogenetic analysis, the *TaRGF5* homeologs showed the highest similarity to *Arabidopsis thaliana RGF5*. Tested RNA-seq data revealed predominantly root-enriched expression for all TaRGF genes, with TaRGF5 exhibiting the most root-preferential and downregulation under drought stress. Quantitative real-time PCR (qRT-PCR) confirmed that drought stress suppressed the expression of *TaRGF5A*, *TaRGF5B*, and *TaRGF5D* in roots of wheat cultivar Sids-13 across all time points, unlike the higher accumulation seen in controls. Promoter analysis predicted a unique BES1 transcription factor binding site exclusively in TaRGF5B, linking brassinosteroid signaling to peptide-mediated root regulation. Structural modeling and molecular docking predicted an interaction between wheat TaRGF5 homeologs and root growth factor-insensitive receptor kinase (TaRGI3), characterized by conserved sulfotyrosine-mediated binding and favorable interaction energetics. Based on this characterization of the wheat RGF gene family, particularly the potential role of TaRGF5 in root development and drought-adaptation signaling, we propose targeting this gene for functional analysis to improve wheat resilience under water-limited conditions.

## 1. Introduction

Wheat (*Triticum aestivum* L.) stands as one of the world’s most vital staple crops. With the global population projected to reach 9.7 billion by 2050, improving wheat productivity and stress resilience is essential to ensuring global food security [1]. However, wheat production faces mounting challenges from climate change, including rising temperatures, soil degradation, and acute water scarcity [2]. Among these, drought stress is one of the most severe environmental constraints, particularly in arid and semi-arid lands where limited, erratic rainfall and high evaporation rates deplete soil moisture during key growth phases [3,4]. This water deficit impairs critical processes such as seed germination [5], root development [6], tillering [7], grain filling [8], and ultimately final yield, often slashing productivity by 20–50% in affected regions [9].

Wheat roots respond to drought by modifying their architecture and physiology to enhance water uptake and minimize water loss [10]. Under water deficit, roots often grow deeper and form more lateral roots and root hairs, thereby increasing total root length and surface area that can forage for water in deeper soil layers [11,12]. This deeper, more extensive root system is associated with traits such as higher root-to-shoot ratios, optimized root length densities, and larger metaxylem vessels, which reduce hydraulic resistance and help sustain plant water status under drought conditions.

Root meristem growth factors (RGFs) are small secreted signaling peptides that regulate plant root development by controlling cell division, cell differentiation, and the overall organization of the root system [13,14,15]. They function as local communication molecules that help roots grow in a coordinated way and respond to internal and external cues such as nutrient availability, water status, and environmental stress [16,17]. In general, root growth factors are important because they help maintain the root meristem, the region where new root cells are produced. By regulating meristem size and activity, they ensure continuous root elongation and proper root architecture [18,19]. These signals also influence the formation of lateral roots and root hairs, which are essential for water and nutrient uptake.

In *Arabidopsis thaliana*, one of the best-studied examples of root growth factors is the *RGF* family, also known as golven (GLV) or CLAVATA3/embryo surrounding region (ESR)-like (CLEL) peptides [20,21]. These peptides are especially important for maintaining the root meristem by promoting the expression of key transcription factors such as PLETHORA 1 and PLETHORA 2 [22,23]. These transcription factors are central regulators of the stem cell niche and root patterning. Arabidopsis RGF peptides function through specific receptor-like kinases at the cell surface, including the AtRGF1 INSENSITIVE receptor family, comprising root meristem growth factor-insensitive receptor kinases (*AtRGIs*) [24]. When the peptide binds to its receptor, it activates downstream signaling pathways that support root stem cell maintenance and developmental responses. This peptide–receptor system is a good example of how plants use small signaling molecules to control complex growth processes. Moreover, root growth factor signaling is tightly linked to other regulatory networks, especially auxin-mediated pathways. This crosstalk helps integrate internal developmental cues with environmental signals, allowing the root system to adjust growth, branching, and patterning in response to changing conditions [15,21,25]. Because of its simple genome, genetic tools, and well-characterized root system, Arabidopsis has become a key model for uncovering how peptide signals shape root development.

In cereals, the RGF peptide pathway functions as a mobile, sulfated signaling module that coordinates root apical meristem activity and lateral root initiation through cognate leucine-rich repeat receptor kinases and downstream auxin and reactive oxygen species (ROS) cascades [15,26]. This positions RGF signaling as a particularly critical regulator in fibrous-rooted crops, where root system architecture is built from multiple root classes, including seminal, crown (nodal), and lateral roots, rather than a single dominant taproot [13]. Because total root length density and soil exploration in such systems emerge from the cumulative output of many short-lived, highly branched axes, even modest RGF-mediated changes in meristem size, cell division rates, or transition-zone dynamics can scale to substantial shifts in root proliferation and branching patterns [13,27,28]. This peptide-based plasticity can contribute to water acquisition by enabling roots to adjust root length density, branching intensity, and rooting depth in response to spatial heterogeneity in soil moisture. However, not all drought-induced morphological changes are inherently adaptive; excessive lateral suppression, premature meristem arrest, or overly steep growth angles may instead reflect stress pathology, hormonal imbalance, or developmental constraints rather than optimized foraging strategies. Moreover, developmental plasticity incurs tangible carbon costs that can penalize shoot growth and yield, and because critical windows for root patterning close once primordia mature, late or severe stress can lock in maladaptive architectures that prove neutral or detrimental in specific edaphic contexts [28,29,30]. Recognizing these trade-offs is essential when considering RGF pathway manipulation as a route to improve drought resilience in cereals.

The wheat genome encodes approximately 4981 putative small secreted peptides (SSPs), of which 1790 could be classified into 38 known peptide families [31,32]. These TaSSP genes are responsive to abiotic stresses such as drought, heat, and salt, and functional annotation suggests roles in stress-related processes, including drought recovery, with families like CLE, C-terminally encoded peptide (CEP), CAP-derived encoded peptide (CAPE), rapid alkalinization factor (RALF), and others enriched among stress-responsive peptides. Importantly, the TaCLE24b peptide was identified as a key regulator in wheat, fine-tuning lateral root development while bolstering drought tolerance [33]. These molecules enhance critical root traits such as increased depth to access subsoil moisture reserves, higher root density for more efficient soil exploration and nutrient scavenging, and architectural plasticity that facilitates dynamic remodeling [34]. Through intricate signaling networks involving receptor-like kinases and transcription factors, they also activate stress-responsive genes; bolster antioxidant enzyme systems (like superoxide dismutase and catalase); modulate hormone crosstalk with abscisic acid (ABA) and auxin; and shield root cells from drought-induced oxidative damage, dehydration, and premature senescence [31,35,36]. Understanding these conserved pathways across wheat genotypes offers promising molecular targets for genetic engineering, marker-assisted breeding, and CRISPR-based editing to develop drought-resilient varieties with superior, adaptive root systems adapted to arid agriculture.

The RGF gene family has not yet been systematically identified in wheat at either the genomic or functional levels. This work aims to fill this gap by mining the wheat genome to identify and characterize the *RGF* family, including the genes, cDNAs, proteins, phylogenetic relationships, and chromosomal distributions. In addition, the expression patterns of *RGFs* in wheat roots and other tissues were further examined, with particular attention to their potential role in root development and stress responses. Finally, the investigation seeks to explore possible interactions between RGF peptides and their putative receptors through protein–peptide docking analysis, providing initial insights into how RGF signaling may operate in wheat root systems.

## 2. Results

### 2.1. Genome-Wide Identification of the TaRGF Gene Family

**Figure 1:**
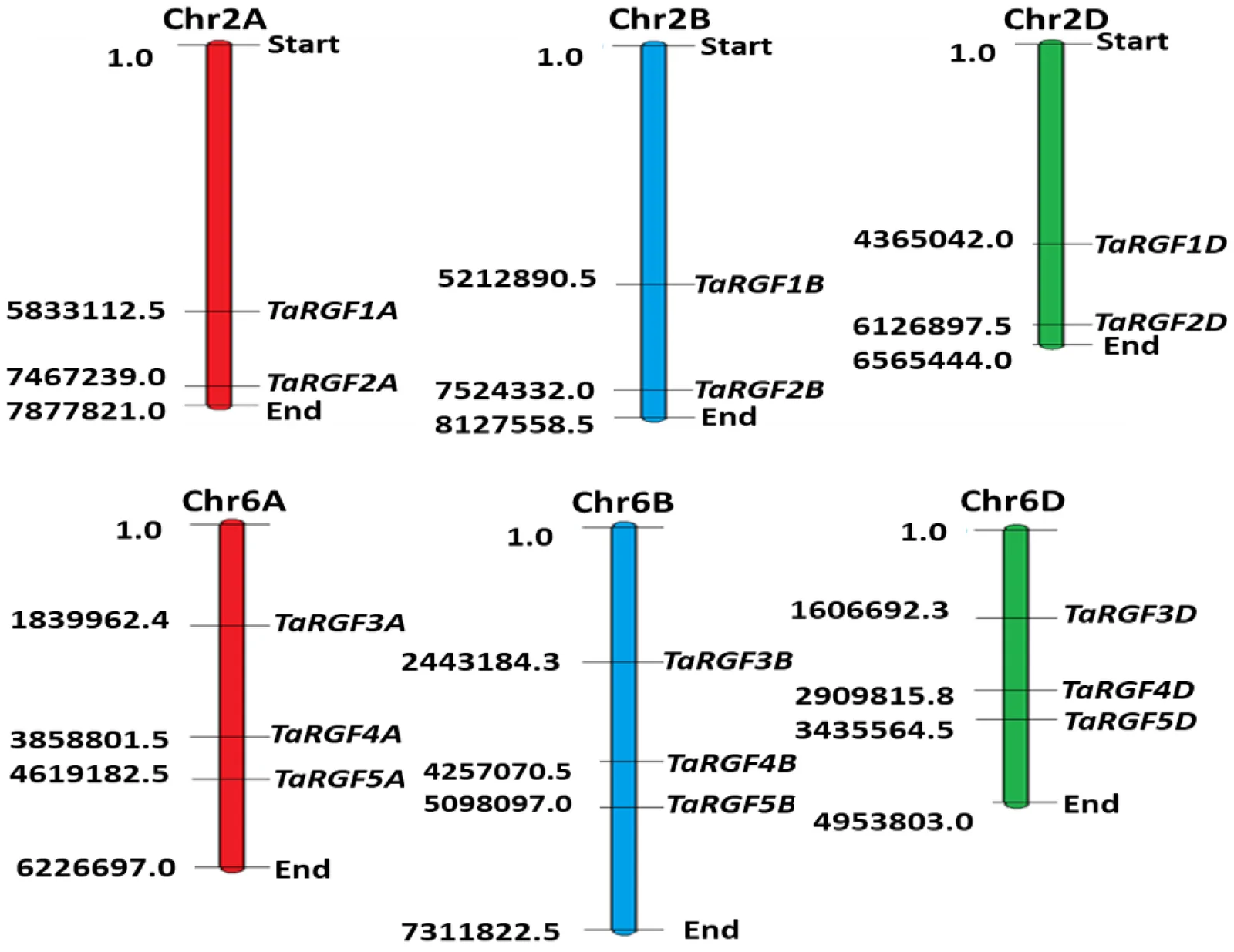
Chromosomal assignment and genomic distribution of *T. aestivum RGFs*. A. Schematic representation of the physical locations of five *RGFs* on the wheat genome. B. Circos plot illustrating the distribution of wheat *RGFs* on chromosomes 2A, 2B, 2D, 6A, 6B, and 6D.

We systematically identified the complete set of RGF gene family members in *T. aestivum* using the latest reference genome, RefSeq version 2.1, assembled by the international wheat genome sequencing consortium (IWGSC). Through this homology-based screening with Arabidopsis *RGFs*, we identified a set of wheat *RGF* candidates, hereafter referred to as TaRGF proteins. Through a genome-wide search, a total of five putative *TaRGFs* were identified, representing the full complement of 15 RGF gene family members assigned to the A, B, and D subgenomes of the hexaploid wheat genome (Data S1). The chromosomal assignment of the *TaRGFs* was determined in the wheat reference genome. The 15 identified *TaRGFs* were mapped to two chromosome groups and were located exclusively on chromosomes 2 and 6 (Figure 1). Each RGF locus was represented by a set of three homeologous copies distributed across the A, B, and D subgenomes, forming the homeologs of *TaRGF1* (2A/2B/2D), *TaRGF2* (2A/2B/2D), *TaRGF3* (6A/6B/6D), *TaRGF4* (6A/6B/6D), and *TaRGF5* (6A/6B/6D). No additional *TaRGFs* were detected on the remaining wheat chromosomes, indicating a highly restricted and non-uniform chromosomal distribution. Gene nomenclature followed the current guidelines for *T. aestivum*, such that homeologous genes were designated according to their chromosome group and subgenome.

**Figure 2:**
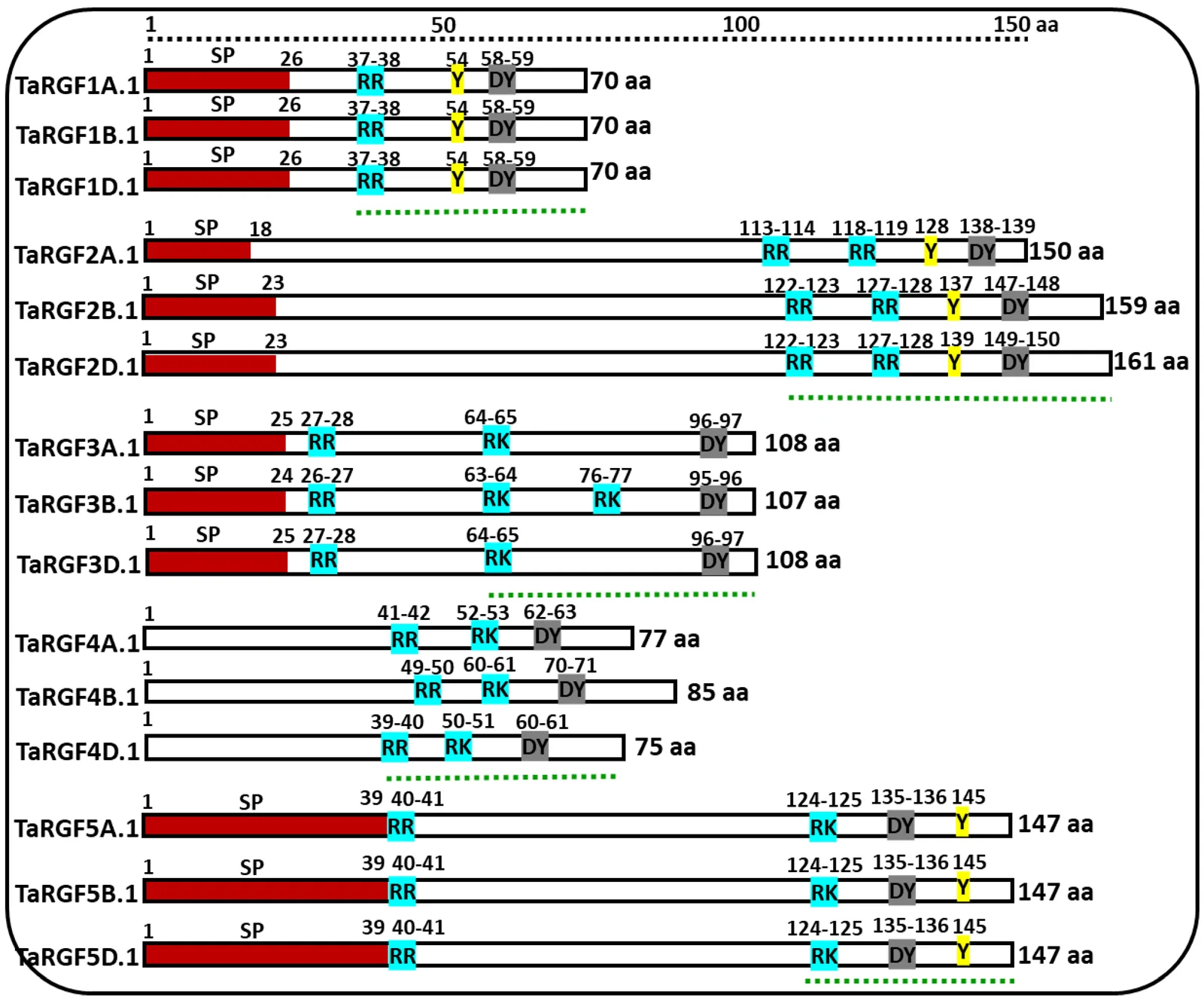
Conserved features of *T. aestivum* RGFs. Schematic representation of RGF precursor protein sequences grouped into five groups (Group 1–5). The N-terminal signal peptides (red bars); RR and RK represent the likely conserved dibasic proteolytic processing sites (cyan boxes); Y indicates conserved tyrosine residues (yellow boxes); DY highlights the Asp–Tyr motif (dark gray boxes); Predicted functional part (green dot line).

At the transcript level, 24 distinct transcript variants were detected, arising from alternative splicing events. Data S2 and Data S3 present the 24 open reading frames (ORFs) and corresponding full-length cDNA sequences of wheat RGF genes, respectively. The full-length amino acid sequences of the TaRGFs were identified (Data S4). All TaRGFs were manually inspected using InterProScan (https://www.ebi.ac.uk/interpro/search/sequence/, accessed on 19 August 2026) and SignalP to confirm the presence of characteristic structural features and to establish a stringent set of typical RGFs. Each member possessed a clearly defined canonical N-terminal signal peptide (red bars), except TaRGF4, which fell below the SignalP probability threshold (Figure 2, Table S1). The N-terminal signal peptides are predicted to mediate secretion of the RGFs into the extracellular space. However, using DeepLoc, a deep-learning-based tool for predicting subcellular protein localization, all TaRGFs were predicted to be localized in the extracellular space (Table S1). Furthermore, the conserved dibasic sites (RR/RK) were identified within the RGF precursor and may serve as recognition motifs for subtilisin-like proteases. These sites are consequently predicted to possibly mediate the proteolytic release of the mature RGF peptide (cyan bars). The DY label highlights the Asp–Tyr motif that contains the tyrosine subjected to post-translational sulfation and required for full RGF biological activity. The predicted mature RGF peptides (green dashed lines) show a high degree of conservation among homeologs and across the five groups. The proteins encoded by the wheat *RGFs* exhibited considerable variation in their basic physicochemical properties. They ranged in length from 70 to 161 amino acid residues, and their predicted molecular masses spanned approximately 8–17 kDa, indicating substantial size diversity within the family. The calculated isoelectric points (pI) extended from 8.5 to 12.1, indicating that these peptides are predominantly basic in nature. In addition, the subcellular localization of the mature protein was predicted by DeepLoc to be secreted to the extracellular space of the cells (Table S1).

**Figure 3:**
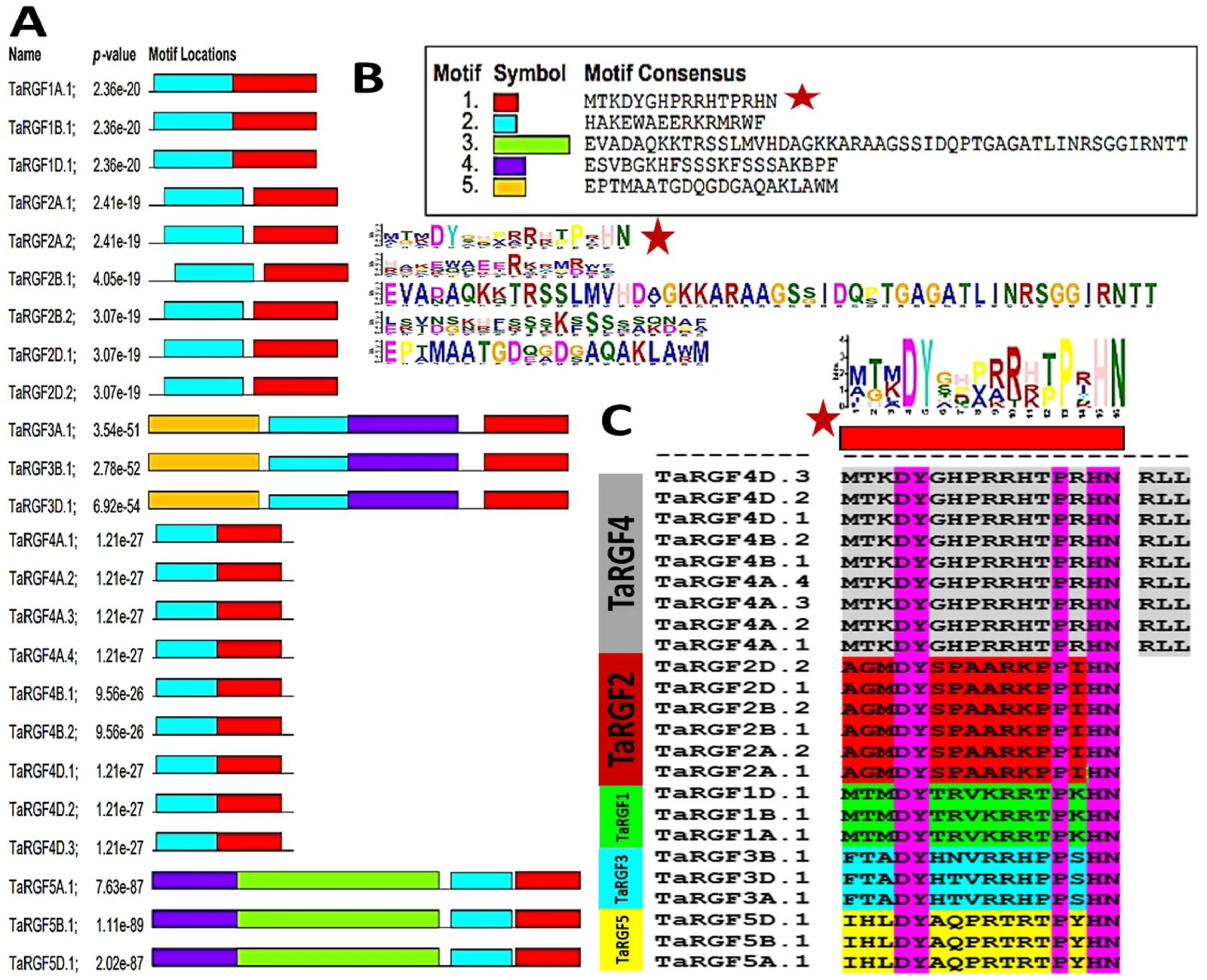
Conserved motif analysis of TaRGFs using MEME analysis. (**A**). The schematic diagram shows the distribution and arrangement of these motifs across each TaRGF. (**B**). The sequence logos of the 5 identified motifs, illustrating amino acid conservation at each position. Predicted functional region (red asterisks). (**C**). The multiple sequence alignment of the TaRGF functional domains.

To investigate the structural conservation of TaRGFs, a conserved motif analysis was performed. Five distinct conserved motifs (motifs 1–5) were identified across the mature TaRGFs (Figure 3A). All five motifs were present in most TaRGFs, although variations in motif order and spacing were observed in some cases. The protein sequences display the amino acid conservation within each motif, where the height of each residue corresponds to its frequency at that position, revealing highly conserved signatures, particularly in motif 1 (Figure 3B). Interestingly, the multiple sequence alignment of the TaRGF functional domain (motif 1) reveals high levels of sequence identity among the three homeologs of each RGF, indicating strong evolutionary conservation (Figure 3C, Table S2). ScanProsite analysis revealed several conserved motifs (Motifs 1, 3, and 4) containing putative post-translational modification and regulatory sites, suggesting potential functional roles in TaRGFs (Table S2). In contrast, Motif 2 and Motif 5 did not match any known Prosite motifs.

### 2.2. Phylogenetic Analysis Reveals Evolutionary Conservation and Diversification of TaRGFs

**Figure 4:**
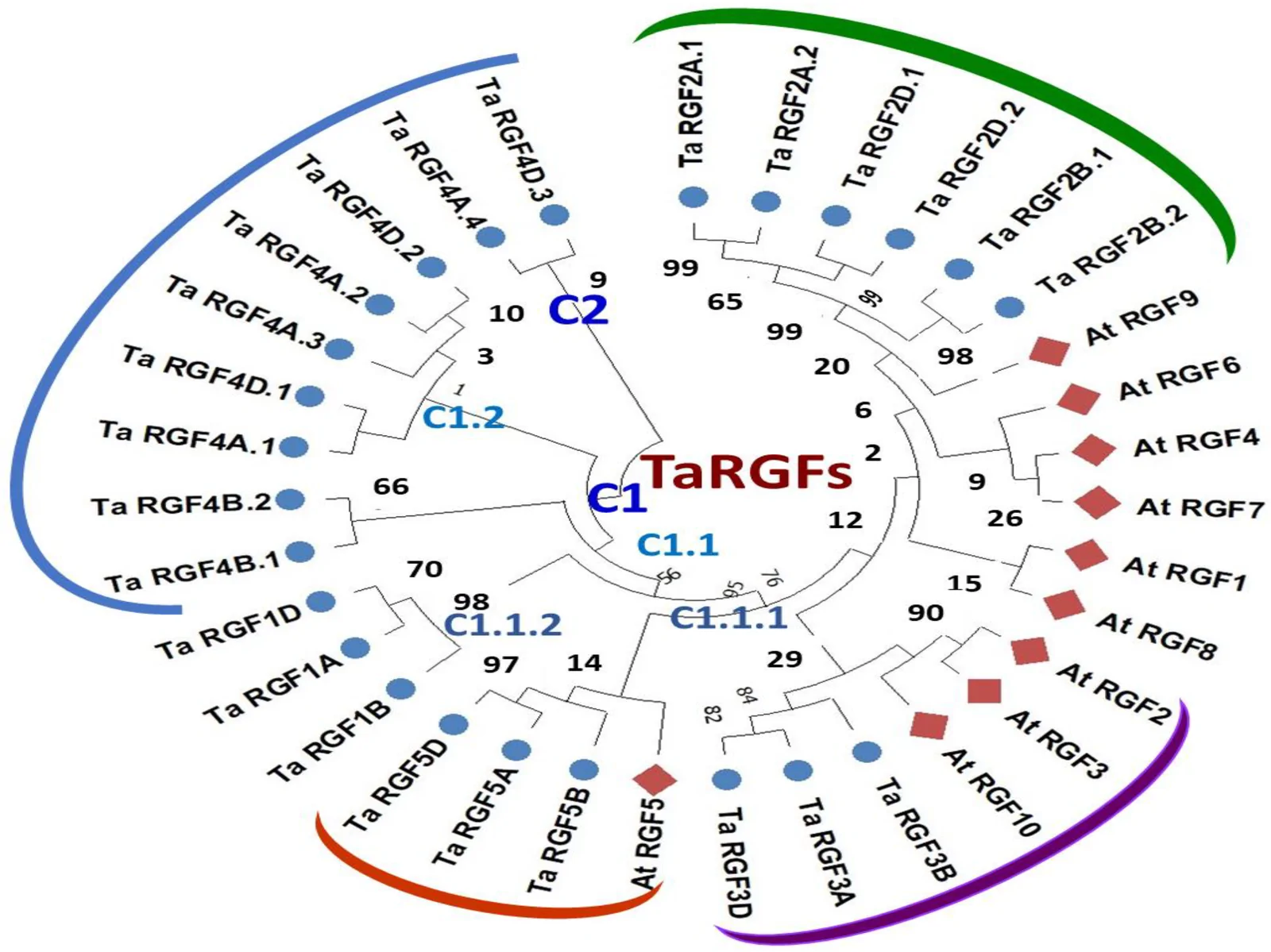
Phylogenetic analysis of *RGFs* from both *T. aestivum* and *A. thaliana*. A circular maximum-likelihood phylogenetic tree was constructed using the amino acid sequences of *TaRGFs* and *AtRGFs*. Wheat TaRGF proteins are indicated by blue circles, whereas *Arabidopsis* AtRGFs are represented by red squares. All *TaRGFs* have a single transcript, written without the splice variant version number. The branch robustness was assessed using 1000 bootstrap replicates. Ta: *Triticum aestivum*; At: *Arabidopsis thaliana*; C: Cluster. TaRGF2 (green curve); TaRGF4 (blue curve); TaRGF5 (red curve).

Phylogenetic analysis was performed using the predicted mature TaRGFs, with the signal peptides excluded. The resulting phylogenetic tree, together with the corresponding *A. thaliana* homologs, revealed two major evolutionary clades (Figure 4). Clade C1 emerged as the largest and most diverse group, comprising two principal subgroups, C1.1 and C1.2. The subclade C1.1 includes both wheat and Arabidopsis TaRGFs. Notably, the three homeologs of TaRGF5 (TaRGF5A, TaRGF5B, and TaRGF5D) grouped with AtRGF5, forming a distinct subcluster that highlights the preservation of conserved domains across species. TaRGF2 members cluster most closely with AtRGF9. In addition, TaRGF3 homeologs clustered closely with AtRGF2, AtRGF3, and AtRGF10. On the other hand, the TaRGF1 homeologs (TaRGF1A, TaRGF1B, and TaRGF1D) clustered together within the subclade C1.1.2. The TaRGF4 isoforms were grouped within clades C1 and C1.2, where multiple transcript isoforms from the A, B, and D genomes clustered tightly together, reflecting strong sequence similarity and possible functional redundancy. Overall, this p phylogeny revealed a pattern of both evolutionary conservation and divergence within the TaRGF family, with certain members maintaining close relationships to AtRGFs, while others exhibit genome expansion.

### 2.3. Tissue-Specific Expression Divergence of TaRGFs

**Figure 5:**
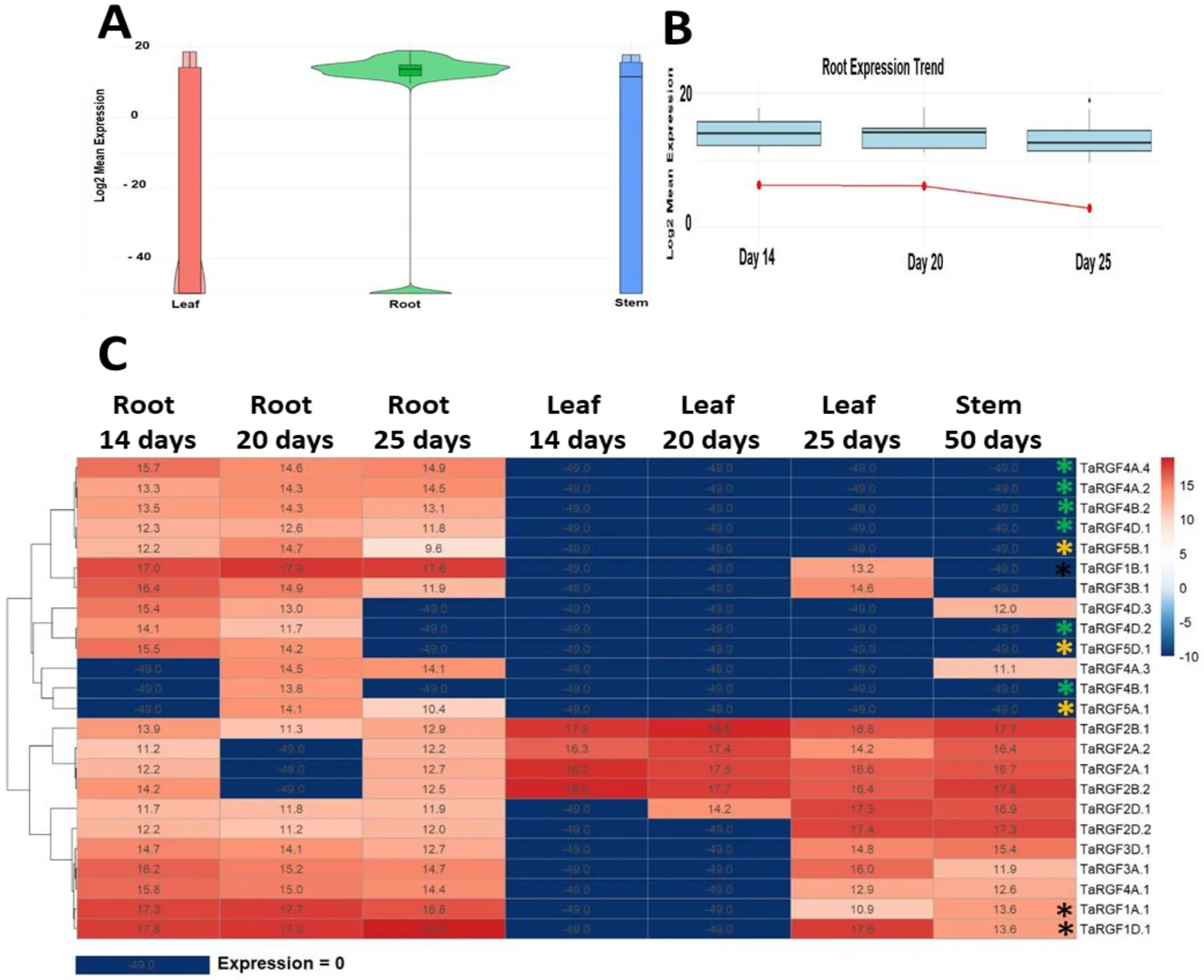
The expression profile of *TaRGFs* in the wheat root, stem, and stem tissues. (**A**). Boxplot showing the overall distribution of log_2_ mean expression levels of all TaRGFs in leaf, root, and stem tissues. (**B**). The log_2_ mean expression levels of TaRGFs in wheat roots at 14, 20, and 25 days old. (**C**). Heatmap showing the transcript abundance of *TaRGF* family across seven wheat libraries, including root, leaf, and stem. Expression values are presented as log_2_-transformed TPM values (gradient red), where dark blue indicates no detectable expression. TaRGF5 homeologs (yellow asterisks); TaRGF4 homeologs (green asterisks); TaRGF1 homeologs (black asterisks).

The gene expression dynamics of the RGF gene family in wheat tissues revealed distinct spatial expression profiles among the members across the examined tissues and stages. Overall, the roots exhibited relatively higher median expression levels compared with leaf and stem tissues (Figure 5A). The analysis of *TaRGF* expression across selected time points in the roots revealed a gradual decline in transcript abundance. The highest expression levels were observed in 14-day-old plants. Subsequently, expression declined at day 20 and further decreased at day 25 (Figure 5B). In contrast, leaf tissues showed the widest variation in expression, reflecting the presence of both highly expressed and transcriptionally silent members, as indicated by expression values of −49.8 (threshold for non-detection). Stem tissues displayed comparatively stable and moderate expression levels across most genes, suggesting a more constitutive expression level.

A separate group of *TaRGFs* showed broad expression across the investigated tissues, indicating that they are not restricted to a single tissue type. These included TaRGF2A.1, TaRGF2A.2, TaRGF2B.1, TaRGF2B.2, and TaRGF2D.1 (Figure 5C). Notably, some of these genes maintained relatively stable expression across the sampled root stages, whereas others showed tissue-dependent variation, implying functional diversification within the *TaRGF* family. The gene-level analysis revealed pronounced tissue-specific divergence among TaRGF homeologs. Several members of the *TaRGF2* group showed clear root expression divergence, whereas members osf *TaRGF1* and *TaRGF3* showed varied expression in leaves with maximal root activity (Figure 5C).

### 2.4. Root-Enriched Expression of TaRGF5

Several TaRGFs showed root-enriched expression, as they were detected in root tissues at 14, 20, and 25 days of age but were undetectable in the examined leaf or stem tissues. A striking observation was the highly root-enriched expression of all members of TaRGF5. This gene was detected exclusively in root tissues in tested libraries, with expression log_2_ values ranging from 12.2 to 15.5 in 14-day-old roots, 14.1 to 14.7 in 20-day-old roots, and 9.6 to 10.4 in 25-day-old roots (Figure 5C). In contrast, TaRGF5 transcript abundance of the three homeologs was absent in all leaf and stem libraries. Similarly, TaRGF4 exhibited a broader but still root-preferential expression profile. TaRGF4 members assigned to the B subgenome showed expression in root stages but were not detected in leaf or stem tissues, supporting their classification as root-enriched candidates. However, unlike TaRGF4B and TaRGF4A.1, both TaRGF4A.3 and TaRGF4D.3 members showed detectable, albeit lower, expression in the leaf and stem libraries (Figure 5C). Moreover, the root-expressed genes exhibited different temporal expression trends during development. Some genes, such as TaRGF4A.4, TaRGF4A.2, TaRGF4B.2, TaRGF4D.1, and TaRGF5B.1, showed higher expression at the early root stages and tended to decrease gradually by 25 days, indicating possible involvement in early root growth. In contrast, other genes showed increased expression over time or maintained relatively stable levels across the three tested root stages.

### 2.5. Drought-Induced Repression of TaRGF5A and TaRGF5B

**Figure 6:**
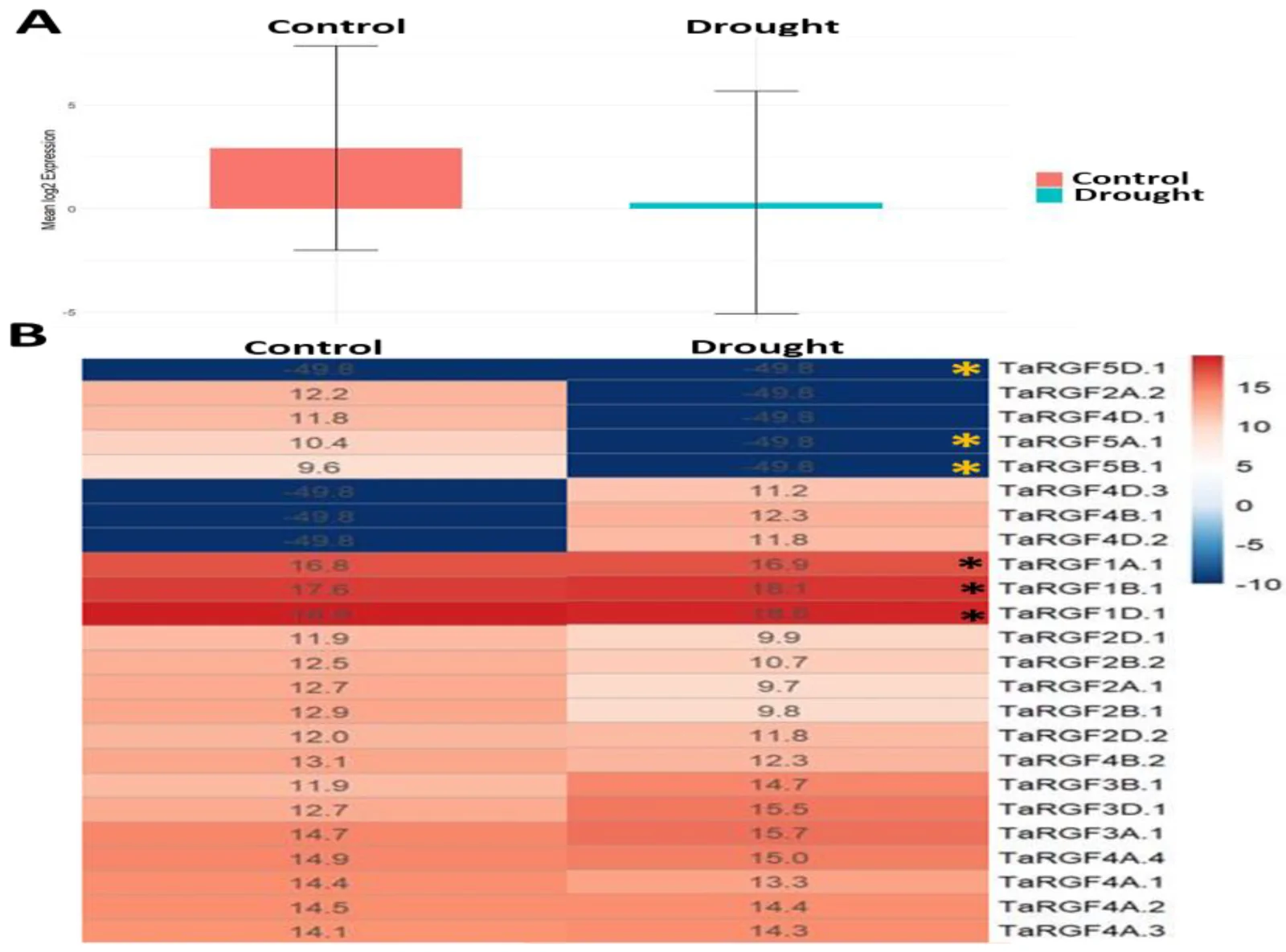
The expression profile of *TaRGFs* in 25-day-old wheat roots under control and drought conditions. (**A**). Overall TaRGF transcript abundance was lower under drought than under control conditions. (**B**). *TaRGF* expression profile: TaRGF5A and TaRGF5D were markedly repressed by drought. Expression values are presented as log2-transformed TPM values (gradient red), where dark blue indicates no detectable expression. TaRGF5 homeologs (yellow asterisks); various TaRGF1 homeologs (black asterisks).

The expression profiles of *TaRGFs* in 25-day-old wheat roots showed clear differences between control and drought conditions. In general, the TaRGF transcript abundance under control was higher than under drought. Despite the variability indicated by the error bars, the overall pattern clearly indicates a decline in average TaRGF expression under drought (Figure 6A). Under control conditions, TaRGF5 members, including TaRGF5A.1 and TaRGF5B.1, displayed moderate to high expression levels, whereas drought treatment caused a marked repression of their expression (Figure 6B). In contrast, the abundance of a subset of transcripts, such as TaRGF4D.3, TaRGF4B.1, and TaRGF4D.2, was induced by drought compared with the control.

Another group of genes remained comparatively stable in expression under both conditions. For example, TaRGF1A.1, TaRGF1B.1, and TaRGF1D.1 exhibited the highest transcript abundance across conditions, with only minor differences between control and drought, suggesting that these genes may be constitutively expressed in roots and less sensitive to water deficit (Figure 6B). Other members, such as TaRGF2D.1, TaRGF2B.2, TaRGF2A.1, TaRGF2B.1, TaRGF2D.2, TaRGF4B.2, TaRGF3B.1, TaRGF3D.1, TaRGF3A.1, TaRGF4A.4, TaRGF4A.1, TaRGF4A.2, and TaRGF4A.3, showed intermediate transcript abundance with modest shifts between the two treatments.

### 2.6. qRT-PCR Validation of TaRGF5 Homeologs Under Drought Stress in Wheat

**Figure 7:**
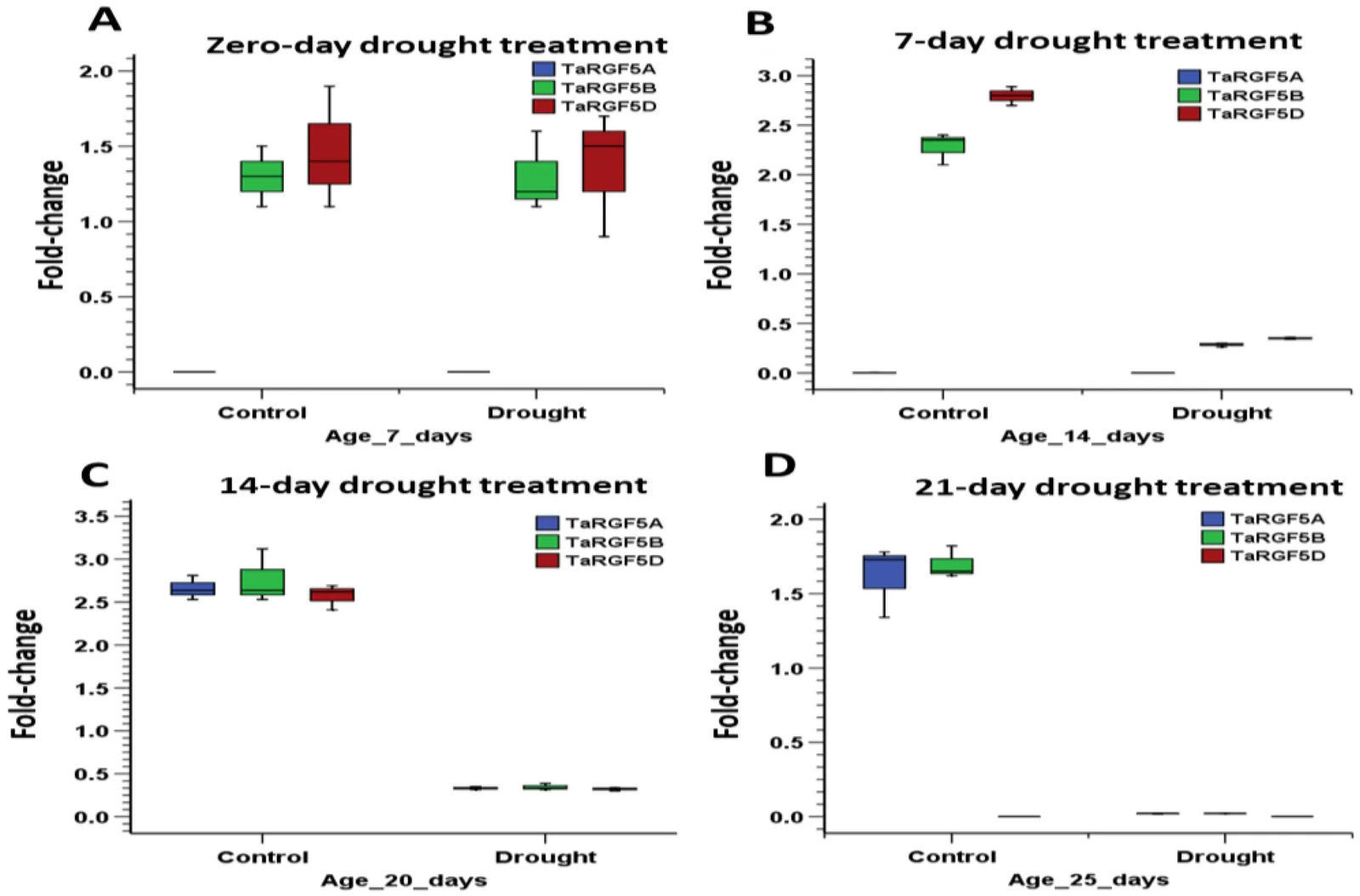
qRT-PCR validation of TaRGF5 homeolog transcript abundance under control and drought conditions in wheat cultivar Sids-13. Relative expression levels of TaRGF5A, TaRGF5B, and TaRGF5D were examined in control and drought-treated plants at different developmental stages. Drought treatment was initiated at 7 days after germination and maintained for 0, 7, 14, and 21 days, as indicated in panels (**A**–**D**). Expression levels were normalized to the internal reference gene translation elongation factor 1α (EF1α) and are presented as fold change. Error bars indicate biological variability among three replicates.

Quantitative real-time PCR (qRT-PCR) analysis was performed to validate the transcript abundance of the three TaRGF5 homeologs in the drought-susceptible wheat cultivar Sids-13 under control and drought conditions. TaRGF5A, TaRGF5B, and TaRGF5D transcript abundance was assessed in roots at 7, 14, 20, and 25 days of plant age, corresponding to 0, 7, 14, and 21 days of drought treatment, respectively, with drought stress initiated at the seedling stage (7 days old). Under control conditions, all three TaRGF5 homeologs showed higher transcript accumulation, whereas drought treatment led to a marked reduction in their expression across the sampled time points. The strongest expression was observed at 20 days in control plants, particularly for TaRGF5A, TaRGF5B, and TaRGF5D, while drought-stressed plants showed consistently low transcript abundance. As drought progressed, transcript abundance of all three homeologs declined sharply. This reduction was most evident after 7, 14, and 21 days of drought treatment, where expression in stressed plants dropped to very low levels, suggesting that drought strongly inhibits TaRGF5 transcription (Figure 7). At zero day of drought treatment, all the plants showed detectable expression of TaRGF5B and TaRGF5D, whereas TaRGF5A remained at very low levels (Figure 7A). After 7 days of drought treatment, the control plants exhibited strong expression of TaRGF5B and TaRGF5D, while TaRGF5A was not substantially expressed. In contrast, drought-treated plants showed only weak residual expression of TaRGF5B and TaRGF5D, with TaRGF5A remaining nearly undetectable (Figure 7B). Following 14 days of drought treatment, control plants maintained relatively high transcript levels for all three TaRGF5 homeologs. However, drought-treated plants displayed a pronounced reduction in expression, with all three genes showing only very low abundance, close to baseline levels (Figure 7C). At 21 days of drought treatment, control plants still showed moderate expression of TaRGF5A and TaRGF5B, whereas TaRGF5D was also low or undetectable. In drought-treated plants, expression of all three homeologs was almost completely suppressed, suggesting a strong inhibitory effect of prolonged drought stress (Figure 7D).

This expression trend was consistent across the sampled time points and confirmed the drought-responsive downregulation observed in the RNA-seq data. Among the three homeologs, TaRGF5B and TaRGF5D generally exhibited stronger transcript abundance than TaRGF5A under control conditions, while drought stress caused a substantial decline in all three transcripts. Overall, the qRT-PCR data corroborate the RNA-seq results and support the conclusion that TaRGF5 homeologs are negatively regulated by drought stress in Sids-13 wheat.

### 2.7. Comparative Prediction of Cis-Regulatory Elements of TaRGFs

**Figure 8:**
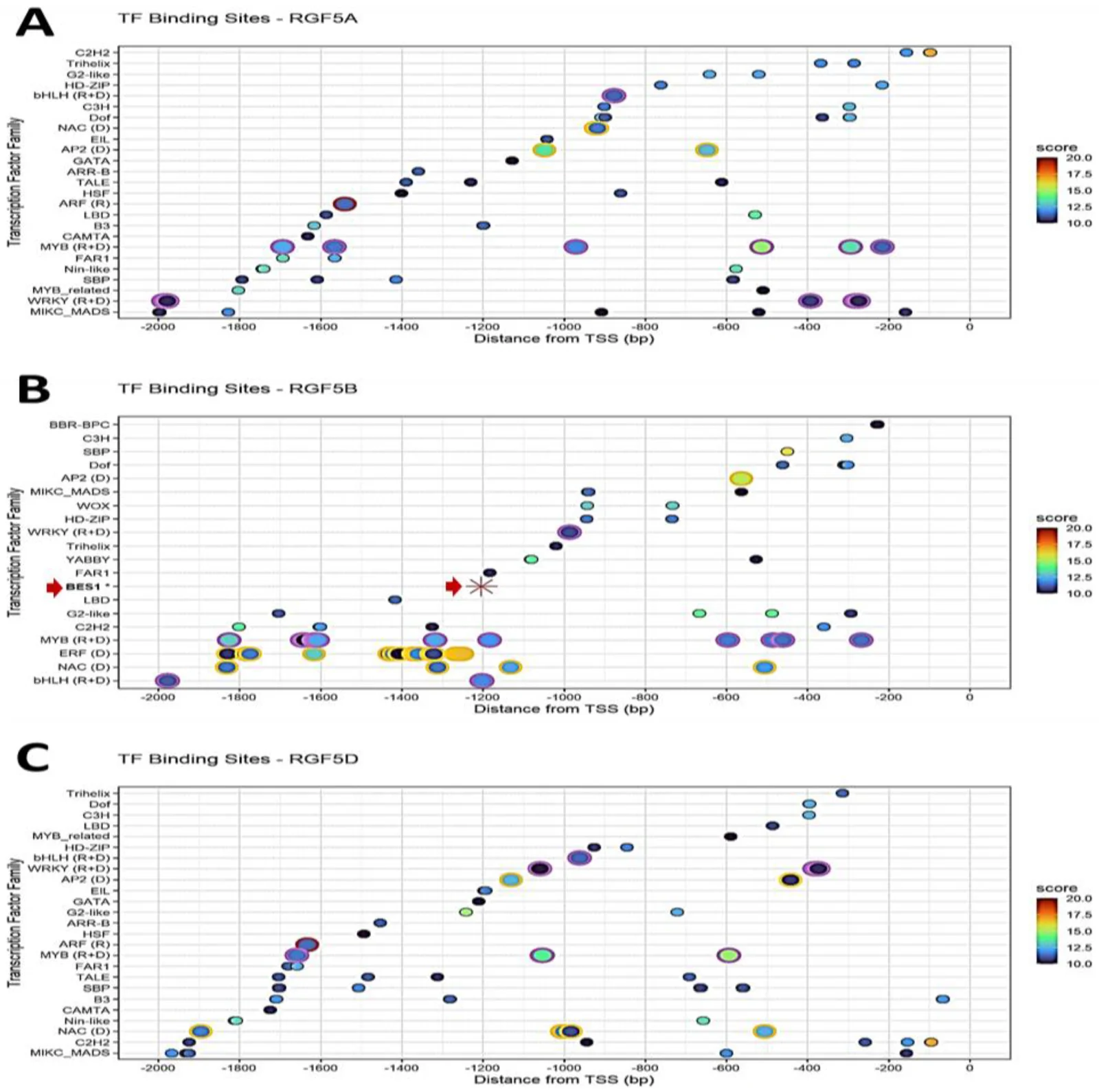
Comparison of predicted promoter architectures of *TaRGF5* homeologs and other *TaRGFs*. Predicted cis-regulatory elements within the 2000 bp upstream regions of the transcription start sites (TSSs) of (**A**). TaRGF5A; (**B**). TaRGF5B; (**C**). TaRGF5D. BRI1-EMS Suppressor 1 element (red asterisk).

To explore why the expression of *TaRGF5* was enriched in root tissues, we investigated the promoter regions of all *TaRGF* family members. The 2000 bp upstream promoter regions of the three *TaRGF5* homeologs (*RGF5A*, *RGF5B*, and *RGF5D*) displayed a shared core set of predicted cis-elements but also revealed clear copy-specific differences from other members (Figure 8). In all three promoters, transcription factor binding sites were distributed across the upstream region and belonged to multiple families associated with growth, hormone response, and stress-related regulation. Notably, these regulatory elements are also conserved in other members of the TaRGF gene family. Notably, *TaRGF5B* was predicted to have a unique BRI1-EMS Suppressor 1 (BES1) cis-element, a motif that was not detected in any other *TaRGFs* (Figure 8B). In addition, *TaRGF5B* revealed a dense cluster of predicted cis-elements in the upstream promoter region, including motifs associated with root regulation, supporting the idea that it may be more strongly or more specifically regulated than the other two homeologs. In contrast, *TaRGF5A* and *TaRGF5D* share common promoter features, but they lack the BES1 element seen in *TaRGF5B*. Their cis-element patterns were still predicted to include motifs related to root development and transcriptional regulation, yet the overall arrangement and composition varied from *TaRGF5B*.

All three TaRGF5 homeologs shared a common core set of transcription factor binding sites with other *TaRGFs*, but each promoter exhibited distinct architectural features. For *TaRGF5A*, the promoter prediction analysis revealed a broad and complex transcription factor binding landscape (Figure 8A). The key transcription factor families included bHLH, MYB, and WRKY, which are associated with root regulation. In contrast, *TaRGF5B* exhibits a more dispersed binding site distribution. Notably, *TaRGF5D* is predicted to present the most compact promoter architecture among the three genes (Figure 8C).

### 2.8. TaRGF5 Likely Functions Through a Leucine-Rich Repeat-like Serine/Threonine Kinase Receptor (TaRGI3)

**Figure 9:**
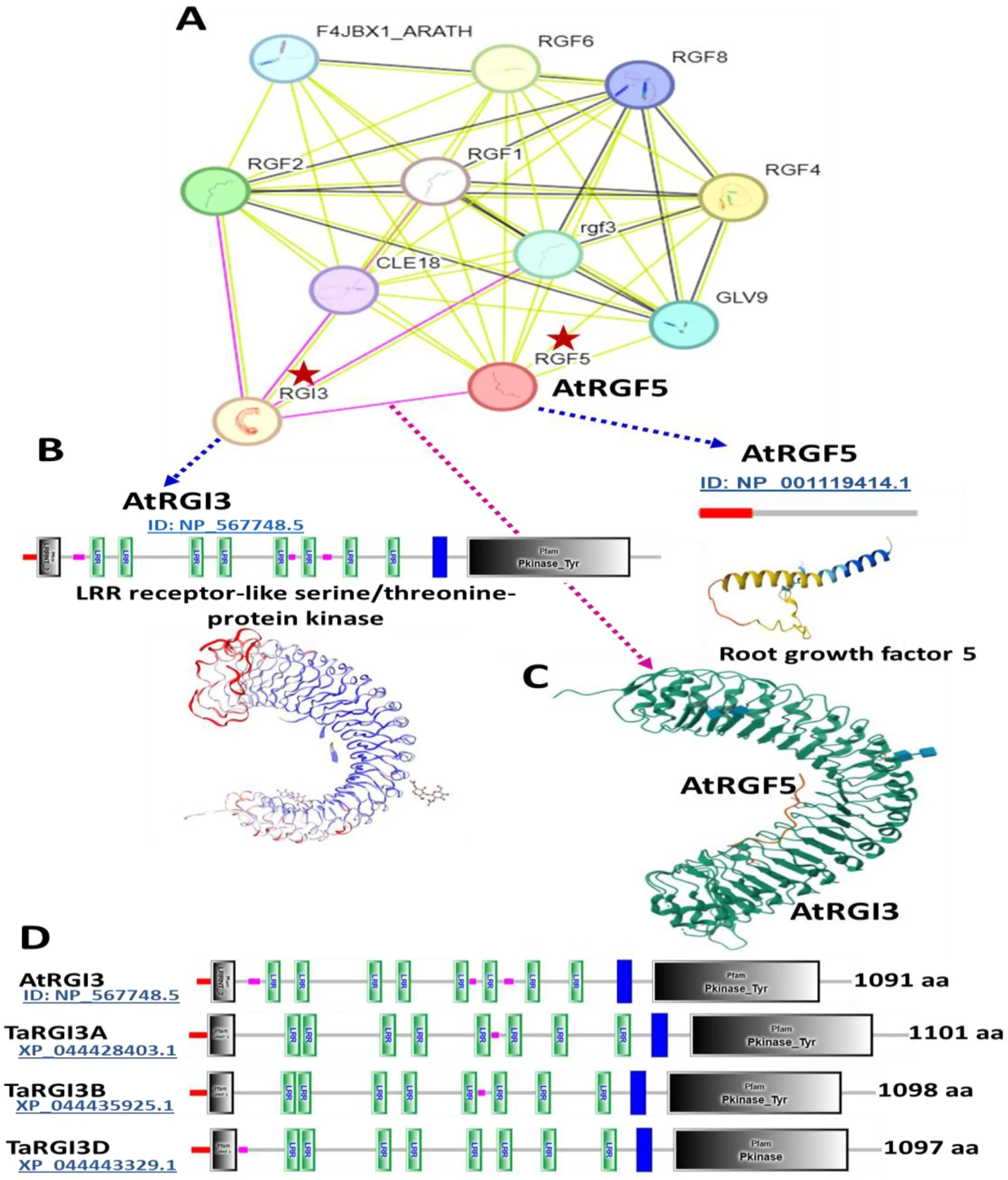
STRING-based protein interaction and comparative structural analyses identify AtRGI3 functions as the putative receptor of AtRGF5. (**A**) STRING protein–protein interaction of Arabidopsis RGF5 and RGI3 (red asterisk) and AtRGI3. (**B**) Domain annotation and structural prediction of AtRGI3 and AtRGF5. (**C**) Molecular interaction modeling further showed stable accommodation of AtRGF5 within the extracellular ligand-recognition pocket (LRR) of AtRGI3. (**D**) Comparative domain architecture of RGI3 in Arabidopsis and wheat exhibiting highly conserved SP (red), LRRNT_2 (gray square), LRR (green), TM (blue), and protein kinase domains (gray rectangle).

Phylogenetic analyses of wheat and Arabidopsis RGFs revealed that TaRGF5 clustered closely with AtRGF5, suggesting evolutionary conservation between these two genes (Figure 4). Given this close evolutionary relationship, TaRGF5 may play a putative functional role similar to that of AtRGF5 in root-associated signaling. This idea was supported by the fact that wheat *TaRGF5* homeologs show enriched expression in roots, suggesting that members of TaRGF5 may play a role in root-associated signaling, just like AtRGF5 (GenBank ID: NP_567748.5). Since peptide hormones often keep the same receptor-binding features as they evolve, the fact that TaRGF5 homeologs together with AtRGF5 suggests that both peptides may share analogous receptor-binding mechanisms. The coexpression association network of AtRGF5 was submitted to the STRING database (https://string-db.org/network/3702.B3H5J1, accessed on 1 March 2026). AtRGF5 exhibited an experimentally validated interaction with the receptor AtRGI3 (NP_567748.5), a leucine-rich repeat-like serine/threonine kinase involved in perceiving AtRGF peptides (Figure 9A). The confidence experimental interaction score (0.9) between AtRGF5 and AtRGI3 indicates that AtRGI3 may function as one of the principal receptor components for this peptide clade. Domain annotation of AtRGI3 demonstrated the presence of multiple extracellular leucine-rich repeat (LRR) motifs followed by a transmembrane helix and an intracellular protein kinase catalytic domain, which together represent the canonical architecture of peptide-sensing LRR receptor-like kinases (Figure 9B). The predicted three-dimensional structure of AtRGI3 formed an extended curve typical of ligand-binding LRR receptors, providing an extracellular recognition surface for small sulfated peptides. In addition, AtRGF5 adopted a compact peptide conformation compatible with extracellular interaction with the receptor LRR domain. Visualization of the modeled AtRGF5–AtRGI3 interaction showed the peptide positioned within the concave binding region of the receptor ectodomain, supporting stable molecular accommodation between ligand and receptor (Figure 9C).

Comparative structural analysis was performed to identify the wheat TaRGI3 protein most closely related to Arabidopsis AtRGI3. The three wheat homeologs, TaRGI3A (XP_044428403.1), TaRGI3B (XP_044435925.1), and TaRGI3D (XP_044443329.1), displayed a highly conserved domain architecture similar to AtRGI3, consistent with their classification as LRR-RLK receptors (Figure 9D). The number and distribution of LRR repeats showed only minor variations, while the general receptor architecture remained strongly preserved, indicating evolutionary conservation of both ligand-binding and kinase signaling functions. This high level of structural similarity supports the possibility that this wheat receptor candidate may represent a bona fide AtRGI3.

### 2.9. Molecular Docking Suggests a Potential TaRGF5–TaRGI3 Interaction

**Figure 10:**
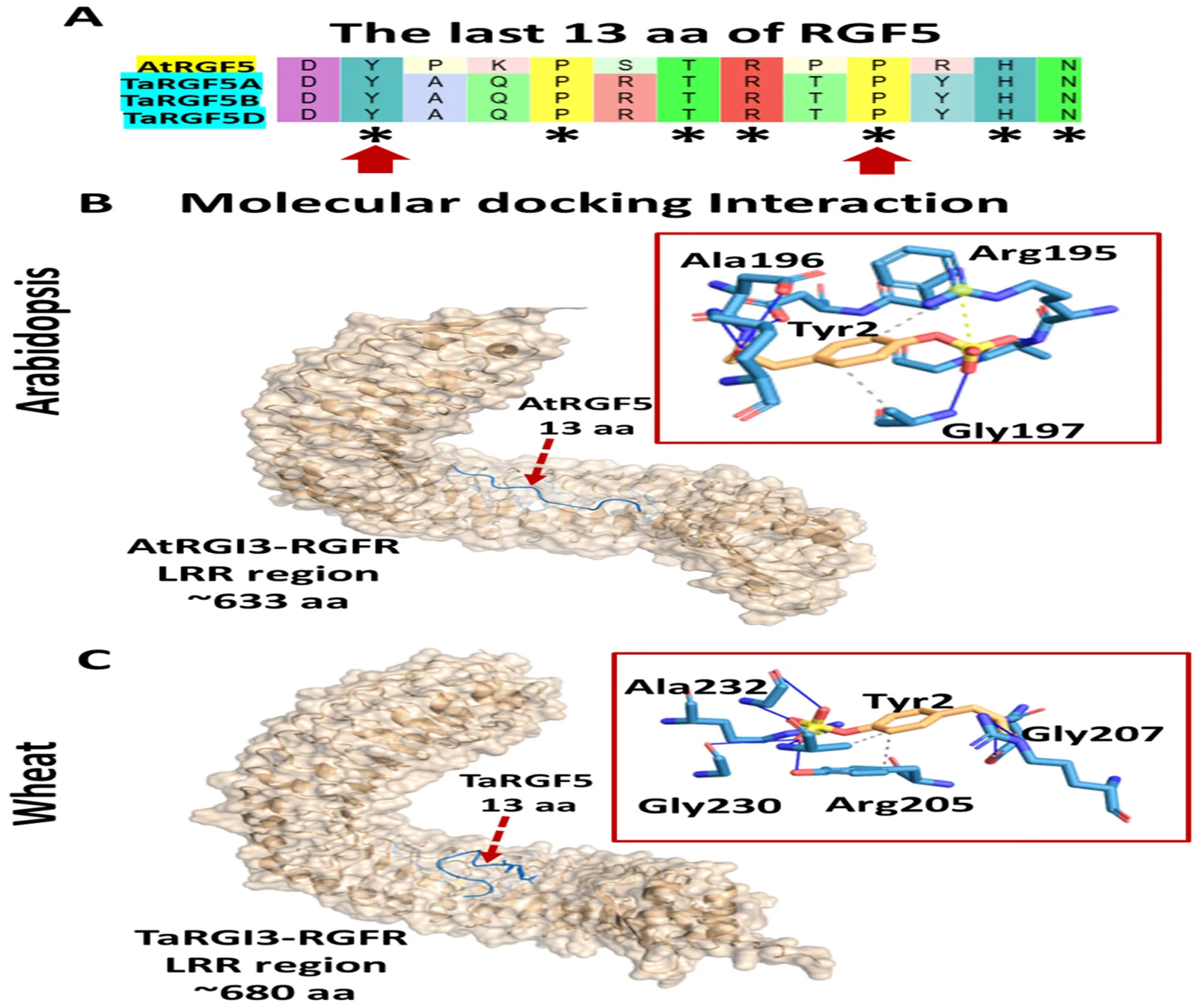
Molecular docking analysis of RGF5 interaction with RGI3 in both Arabidopsis and wheat. (**A**). The mature 13-amino-acid Arabidopsis and wheat RGF core peptides defined by the conserved motifs. Conserved residues between Arabidopsis and wheat RGF1 are shown with black asterisks. (**B**). Arabidopsis reference AtRGF5–AtRGI3 interaction. (**C**). Wheat TaRGF5–TaRGI3 interaction.

To investigate the molecular basis of wheat RGF5 recognition by RGI3, a molecular docking strategy was implemented using the HADDOCK (https://wenmr.science.uu.nl/haddock2.4/, accessed on 19 August 2026) server. Based on the crystallographic model of the Arabidopsis RGF5-RGI3 interaction (PDB ID: 5HZ3), the mature ligand peptide was identified as a 13-amino-acid sequence located at the C-terminal region of RGF5 (Figure 10A). Sequence alignment of this region across Arabidopsis and the three wheat homeologs revealed a highly conserved motif comprising aspartic acid (D), tyrosine (Y), alanine (A), glutamine (Q), proline (P), arginine (R), threonine (T), histidine (H), and asparagine (N) (black asterisks). Notably, the mature peptides (13 aa) of all wheat homeologs were found to be identical. Consequently, one homeolog of the mature peptides was subsequently structurally modeled. Key post-translational modifications (PTMs), including sulfation of Tyr2 (TyrS2) and hydroxylation of Pro10 (HyPro10), were introduced (Figure 10A). Both PTMs are known to be essential for RGF biological activity. The analysis of the Arabidopsis RGI3 receptor indicated that the extracellular LRR domain (residues 24–700) constitutes the principal peptide-binding interface. Accordingly, ligand–receptor docking for both the Arabidopsis and wheat systems was restricted to the modeled LRR ectodomain for interaction analysis. In addition, the LRR domains of the three TaRGI3 homeologs were structurally modeled and aligned using PyMOL, and their pairwise structural similarity was evaluated based on the root-mean-square deviation (RMSD) of atomic coordinates. The analysis revealed a high degree of structural conservation among the three homeologs, with RMSD values below 0.3 Å, indicating near-identical backbone conformations. Based on this, one model was selected as a representative structure for subsequent molecular docking analysis.

The Arabidopsis reference interaction (AtRGF5–AtRGI3) yielded a HADDOCK score of −202.4 ± 1.2, with van der Waals, electrostatic, and desolvation contributions of −71.7 ± 5.3, −624.8 ± 42.2, and −8.5 ± 2.1, respectively. By comparison, docking of the wheat TaRGI3 model with the same peptide yielded a HADDOCK score of −105.3 ± 2.8. Although this score was less favorable than that obtained for the Arabidopsis reference complex, the docking results indicate that the TaRGI3 model may accommodate the TaRGF5 peptide within a compatible binding interface. The modeled wheat TaRGF5–TaRGI3 complex exhibited a van der Waals energy of −27.8 ± 2.5, an electrostatic energy of −397.7 ± 18.0, and a desolvation energy of −2.5 ± 1.0 (Table 1). These energy components provide additional computational support for the potential compatibility of the TaRGF5 peptide with the TaRGI3 binding interface.

Residue-level analysis indicated that peptide recognition in both species involved the sulfated tyrosine (TyrS) residue, consistent with the established importance of this modification in Arabidopsis. In the wheat model, TyrS2 was positioned within the predicted TaRGI3 binding interface (Figure 10C), forming a salt bridge with Arg205 and hydrogen-bond interactions with Gly207, Gly230, and Ala232, as well as hydrophobic contacts involving Ala232 and Tyr11. The observation of a similar TyrS-centered interaction pattern in the wheat and Arabidopsis models suggests that sulfation-dependent peptide recognition may be structurally conserved.

### 2.10. Molecular Dynamics Simulations Reveal the Potential TaRGF5–TaRGI3 Interaction

**Figure 11:**
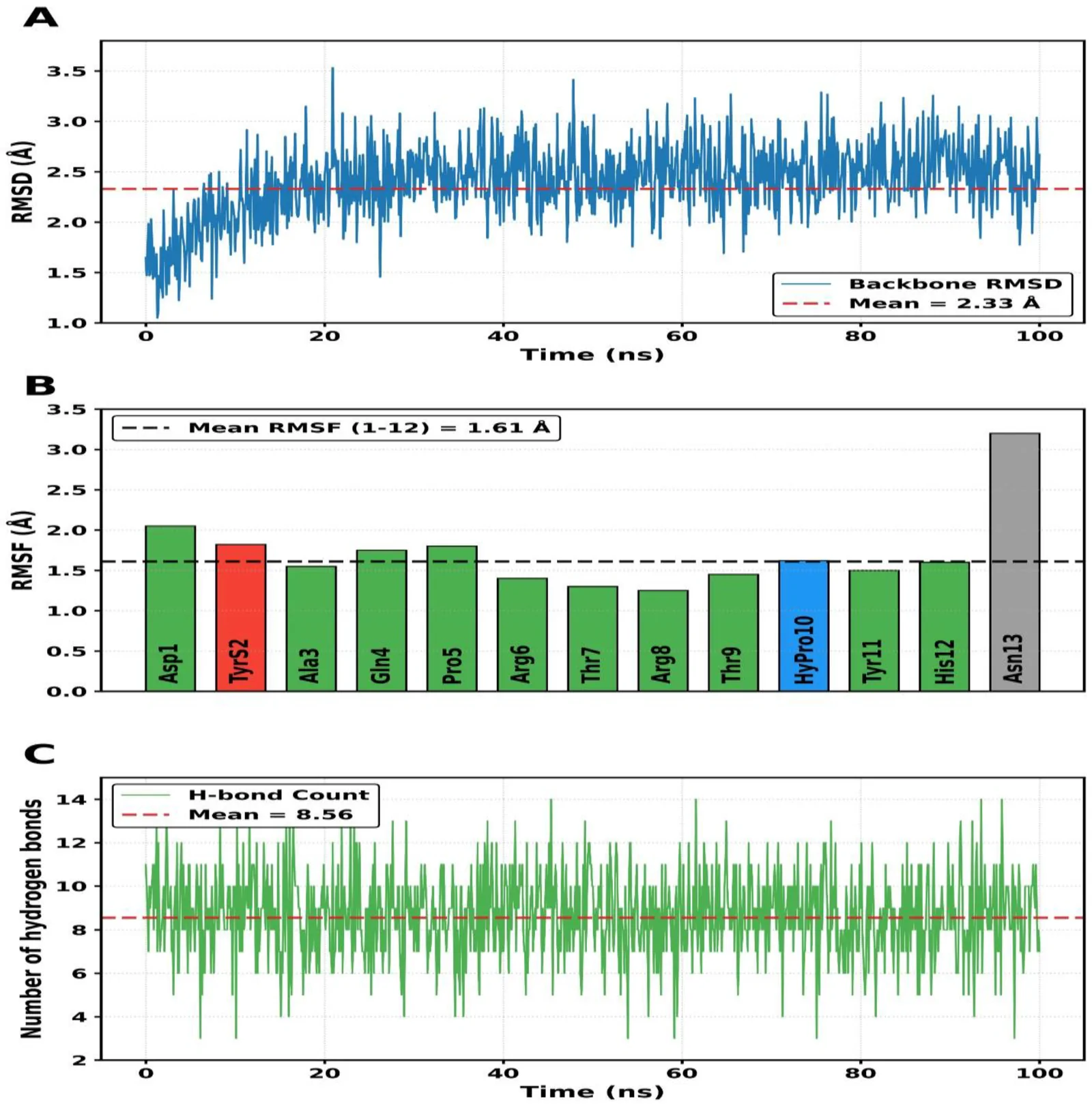
Molecular dynamics analysis of the TaRGF5–TaRGI3 interaction. (**A**). Backbone RMSD of the TaRGF5–TaRGI3 interaction over a 100 ns simulation. (**B**). Residue-level flexibility (RMSF) of the TaRGF5 peptide. (**C**). Intermolecular hydrogen bonds between TaRGF5 and TaRGI3 during the simulation.

Molecular dynamics simulations demonstrated that the TaRGF5–TaRGI3 interaction maintained a stable interaction, indicating strong structural integrity and persistent ligand–receptor engagement throughout the 100 ns trajectory (Video S1). This stability was further characterized using backbone RMSD, residue-level flexibility (RMSF), and intermolecular hydrogen bond analyses. The backbone RMSD exhibited an initial equilibration phase within the first 30 ns, after which the complex stabilized, maintaining an average RMSD of 2.33 ± 0.41 Å, with values ranging from 1.24 to 3.54 Å (Figure 11A). During the final third of the simulation, the RMSD plateaued at approximately 2.51 Å, with no evidence of abrupt conformational shifts, peptide dissociation, or unbinding events, indicating sustained structural integrity throughout the trajectory. Residue flexibility analysis of the 13-residue TaRGF5 peptide revealed generally low atomic fluctuations, with a mean RMSF of 1.61 Å when excluding the terminal residue (Figure 11B). The post-translationally modified residues, sulfotyrosine (TyrS2) and hydroxyproline (HyPro10), exhibited RMSF values of 1.82 Å and 1.62 Å, respectively, supporting conformational stability during receptor binding. In contrast, the C-terminal residue Asn13 showed elevated flexibility (RMSF = 3.20 Å), consistent with its solvent-exposed position.

Intermolecular hydrogen bond analysis indicated the persistence of peptide–receptor contacts throughout the molecular dynamics simulation (Figure 11C). Applying a donor–acceptor distance cutoff of 3.5 Å and an angular criterion of 30°, the complex maintained an average of 8.56 ± 1.90 hydrogen bonds per frame across 1000 analyzed frames, with values ranging from 3 to 14. The distribution was consistently centered between 6 and 11 hydrogen bonds, and no frame lacked intermolecular hydrogen bonding, indicating continuous receptor engagement. Together, these results indicate that the modeled TaRGF5–TaRGI3 complex maintained a relatively stable conformation throughout the 100 ns molecular dynamics simulation. Notably, the electrostatic interaction between the sulfated TyrS2 residue and the guanidinium group of Arg205 was maintained throughout the simulation, with an occupancy of 100%, a mean interaction distance of 4.81 Å, and a minimum distance of 4.17 Å. These observations support the persistence of the modeled TyrS2–Arg205 interaction and are consistent with a stable predicted peptide–receptor interface.

## 3. Discussion

Wheat is a cornerstone of global food security, yet its productivity is increasingly threatened by drought stress, which severely impairs root development and water uptake. RGFs are small secreted peptides that act as master regulators of root meristem maintenance, stem cell niche specification, and lateral root formation in model plants such as *A. thaliana* [15,20,21]. Despite the established importance of RGF signaling in root development and stress adaptation, the RGF gene family has remained completely uncharacterized in the complex wheat genome. To date, no systematic identification, expression profiling, or receptor interaction analysis has been performed for *TaRGFs*. This knowledge gap hinders the translation of fundamental insights from model systems to wheat improvement. Given that wheat root architecture is critical for drought tolerance and that peptide signals offer promising targets for precision breeding, there is an urgent need to identify the wheat RGF complement, understand their expression patterns in roots under normal and water-limited conditions, and predict their cognate receptors through computational approaches. The present study addresses these gaps by providing the first genome-wide analysis of the *TaRGF* family.

A total of 15 *TaRGFs* were identified in the A, B, and D subgenomes of wheat and classified into five homeologous groups (*TaRGF1*–*TaRGF5*) located exclusively on chromosomes 2 and 6. Unlike other small secreted peptide families in wheat, which are more broadly distributed across the genome, the TaRGF family exhibited restricted chromosomal localization. Each group contained three corresponding homeologs, consistent with the hexaploid structure of wheat, suggesting limited gene duplication or loss after polyploidization. All TaRGFs except TaRGF4 possessed canonical N-terminal signal peptides, supporting their predicted secretion into the apoplast. The identification of conserved dibasic processing sites (RR/RK) immediately upstream of the DY motif aligns with the established maturation pathway of RGF peptides, which require subtilisin-like protease-mediated cleavage to release the biologically active C-terminal peptide [37,38,39,40]. Notably, motif analysis revealed that the functional domain corresponding to the mature peptide (motif 1) contained putative phosphorylation sites for protein kinase and protein kinase C, suggesting that TaRGF activity may involve additional layers of post-translational regulation beyond the well-characterized tyrosine sulfation and proline hydroxylation events. The presence of these modification sites within the mature peptide region is particularly intriguing, as it implies that receptor binding affinity or downstream signaling intensity could be dynamically tuned by cellular kinase activity in response to environmental cues [41,42].

The phylogenetic analysis partitioned TaRGFs into two primary clades, with TaRGF5 members clustering robustly with AtRGF5. This close evolutionary relationship, combined with the root-enriched expression of TaRGF5 homeologs, suggests potential functional similarity between wheat and Arabidopsis. In Arabidopsis, AtRGF5 acts as a key regulator of root meristem maintenance by promoting PLETHORA transcription factor expression [13,21,43]. By analogy, TaRGF5 may play a similar role in wheat roots, making it an attractive target for genetic improvement of root system architecture. Interestingly, TaRGF2 homeologs clustered with AtRGF9, while TaRGF3 grouped with AtRGF2/3/10, suggesting that the ancestral RGF gene family diversified into potentially functionally distinct subclades prior to the divergence of monocots and dicots. The absence of wheat members clustering with AtRGF1, AtRGF6, or AtRGF7/8 suggests either lineage-specific gene loss in Poaceae or functional redundancy compensated by other family members.

RNA-seq analysis revealed that *TaRGFs* exhibit tissue variation in their expression, with the highest transcript accumulation observed in root tissues. This root-preferential expression was consistent with the known role of RGF peptides in regulating root apical meristem maintenance and lateral root development [20,41]. Among the identified genes, TaRGF5 homeologs showed high expression in roots and were undetectable in other tested tissue libraries, suggesting that TaRGF5 homeologs may function as root-enriched genes with a specialized role in the roots. To provide independent support for the RNA-seq observations, the expression profiles of selected TaRGF5 homeologs were validated by qRT-PCR using independently analyzed biological samples. The qRT-PCR results showed expression trends consistent with those detected in the RNA-seq datasets, including the preferential expression of TaRGF5 in roots and its suppression under drought conditions. This agreement between two complementary approaches strengthens the biological plausibility and reproducibility of the identified expression patterns. qRT-PCR analysis demonstrates that drought stress consistently suppresses all three TaRGF5 homeologs in wheat cultivar Sids-13 across all time points, with expression peaking at 20 days in controls but dropping to near-undetectable levels under prolonged drought. These findings suggest that TaRGF5 homeologs may represent a promising candidate for further investigation toward enhancing drought resilience in wheat.

In contrast, *TaRGF1* and several *TaRGF4s* displayed broader expression profiles across multiple tissues, indicating possible functional diversification within the family. A progressive reduction in *TaRGF* expression from day 14 to day 25 was observed in roots, which may reflect the decline in meristematic activity during root maturation, consistent with the established function of RGF peptides in sustaining meristem size and cell proliferation. Differences in temporal expression among TaRGFs further suggest functional specialization. For example, the abundance of TaRGF4A.4 and TaRGF5B.1 was highly expressed during early stages before gradually decreasing, implying roles in early root establishment, whereas other genes maintained relatively stable expression, possibly contributing to general root growth.

One of the most striking findings of this study is the marked repression of *TaRGF5* homeologs’ expression under drought stress in 25-day-old wheat roots. This observation is counterintuitive at first glance, as one might expect root growth-promoting peptides to be upregulated to enhance root foraging capacity under water deficit. However, drought-induced inhibition of root growth is a well-documented adaptive strategy that redirects limited carbon resources to shoot survival and reproduction when water availability is severely limited [44,45,46]. Repression of TaRGF5 under drought may therefore represent a regulatory mechanism to slow root meristem activity, reduce metabolic demand, and prioritize resource allocation to existing root structures that can still access remaining soil moisture. This interpretation is consistent with the observation that Arabidopsis CLEs, another root-regulatory peptide in wheat, modulate lateral root development in a context-dependent manner during drought stress [47]. In contrast to *TaRGF5* homeologs, *TaRGF1* homeologs maintained constitutively high expression under both control and drought conditions, suggesting that these family members perform fundamental root functions that must be sustained even under stress. Similarly, *TaRGF4D.3*, *TaRGF4B.1*, and *TaRGF4D.2* were induced by drought, indicating that certain RGFs may actively promote specific root adaptive responses, such as increased root hair density or altered xylem differentiation, under water-limited conditions [48]. This functional diversification within the *TaRGF* family underscores the complexity of peptide-mediated root regulation and suggests that different family members contribute to distinct aspects of root development and stress adaptation.

The comparative promoter analysis revealed that all *TaRGF5* homeologs shared core cis-regulatory elements with other family members, consistent with their common evolutionary origin. However, the identification of a BES1 transcription factor binding site was exclusively predicted in the *TaRGF5B* promoter. BES1 was involved in the regulation of brassinosteroid signaling that integrates hormonal and environmental signals to control plant growth and stress responses [49,50]. The presence of this element suggests that *TaRGF5B* expression may be directly regulated by brassinosteroid signaling, potentially linking steroid hormone pathways with peptide-mediated root development. This finding aligned with emerging evidence for crosstalk between RGF signaling and other hormonal pathways, particularly auxin, in coordinating root growth [21]. The absence of this element in *TaRGF5A* and *TaRGF5D* raised the possibility of subgenome-specific regulatory divergence, whereby the B homeolog acquired a unique mode of transcriptional control that may confer distinct expression dynamics or stress responsiveness. In addition, the homeolog-specific distribution in *TaRGF5* raises the possibility of dosage effects, subfunctionalization, or broader regulatory divergence among the *TaRGF5A*, *TaRGF5B*, and *TaRGF5D* promoters. If the three homeologs are expressed at different levels or under distinct hormonal and environmental conditions, their activity may contribute to the divergence of *TaRGF5* functional properties for normal root-associated signaling. Alternatively, individual homeologs may have undergone partial subfunctionalization, with *TaRGF5B* responding more strongly to brassinosteroid-related signals while *TaRGF5A* and *TaRGF5D* retain other regulatory inputs. These remain hypothetical, however, because the presence of a predicted cis-regulatory element does not demonstrate its in vivo functionality or establish differences in homeolog-specific expression. Experimental analyses, including homeolog-resolved expression profiling and promoter-reporter or BES1-binding assays, will be required to determine whether these regulatory differences have functional consequences.

The close phylogenetic clustering of TaRGF5 with AtRGF5 provided the initial basis for hypothesizing that TaRGF5 signals through homologous receptors of the Arabidopsis RGI3 family. This evolutionary relationship is functionally meaningful, as homologous peptide–receptor pairs may retain ligand–receptor compatibility across large phylogenetic distances due to strong selective pressure on peptide-binding interfaces [51,52]. The three wheat TaRGI3 homeologs exhibited highly similar backbone conformations, with pairwise RMSD values below 0.3 Å, indicating structural conservation among the modeled proteins. This structural similarity suggests that the LRR ectodomain and its predicted ligand-binding region may be conserved across the three subgenomes. Accordingly, the TaRGI3 homeologs may possess similar structural capacities for TaRGF5 recognition; however, this possibility remains to be experimentally validated. If confirmed, such conservation could potentially contribute to functional redundancy among TaRGI3 homeologs in hexaploid wheat.

Molecular docking using HADDOCK provided computational evidence for a direct TaRGF5–TaRGI3 interaction. The wheat TaRGF5–TaRGI3 complex yielded a HADDOCK score of −105.3 ± 2.8, whereas the Arabidopsis reference complex produced a score of −202.4 ± 1.2. Although the wheat model showed a less favorable numerical score, HADDOCK scores are most appropriately interpreted for relative ranking among models generated under comparable conditions. These results provide computational evidence supporting the structural plausibility of a TaRGF5–TaRGI3 interaction. Several non-mutually exclusive explanations may account for this score difference. First, the Arabidopsis reference complex was derived from a crystallographically determined structure, whereas the wheat model is computationally predicted, and small inaccuracies in side-chain placement within the binding pocket may reduce the calculated docking score. Second, the wheat LRR ectodomain model was trimmed to remove low-confidence regions (pLDDT < 50), which, while improving structural reliability, may have excluded flexible loops that contribute favorably to ligand accommodation in the native protein. Despite these caveats, the consistent application of the same docking protocol to both systems allows for meaningful comparative interpretation, and the wheat score remains well below the threshold for non-specific interactions.

Additional support for a conserved signaling mechanism comes from the residue-level interaction analysis. In both Arabidopsis and wheat, the sulfated tyrosine at position 2 (TyrS2) appears to function as the primary molecular anchor, forming a salt bridge with a conserved arginine (Arg195 in AtRGI3; Arg205 in TaRGI3) and establishing a network of hydrogen bonds with surrounding backbone amides and side chains. This sulfotyrosine–arginine interaction may be a recurring theme in peptide hormone recognition by LRR-RLKs, observed not only in RGF–RGI systems but also in related peptide–receptor pairs such as PSK–PSKR and PSY–PSYR [40,53,54,55]. The conservation of this anchoring mechanism across ~160 million years of evolutionary divergence between monocots and dicots suggests its functional indispensability [56]. Tyrosine sulfation is catalyzed by tyrosylprotein sulfotransferases (TPSTs) in the Golgi apparatus, and mutations that abolish either the sulfation site or the cognate receptor arginine completely disrupt RGF signaling in Arabidopsis [57,58]. The presence of a conserved TyrS2-centered interaction in the wheat model raises the possibility that TaRGF5 recognition may similarly depend on tyrosine sulfation, potentially involving TPST-mediated modification as reported for RGF peptides in Arabidopsis. However, this mechanism has not yet been experimentally validated in wheat. Future functional studies examining TaRGF5 sulfation and TPST activity will be necessary to determine whether this modification contributes to TaRGF5-mediated signaling and root developmental responses in wheat.

The 100 ns molecular dynamics simulation provided support for the structural stability and potential biological relevance of the TaRGF5–TaRGI3 interaction. The backbone RMSD stabilized at 2.33 ± 0.41 Å after the initial equilibration phase, with no detectable peptide dissociation or major conformational rearrangements throughout the trajectory. This level of stability is comparable to experimentally validated peptide–receptor interactions, such as the Arabidopsis RGF5–RGI3 crystallographic structure, and reinforces the reliability of the predicted binding mode derived from docking. The sustained intermolecular hydrogen bonding network, averaging 8.56 bonds per frame with continuous presence throughout the simulation, demonstrates robust and persistent peptide–receptor engagement. Most critically, the sulfotyrosine (TyrS2)–Arg205 electrostatic interaction maintained 100% occupancy across all frames, with a stable interaction distance of 4.81 Å. This persistent salt bridge supports the idea that the sulfotyrosine–arginine interaction is a key determinant of complex stability and may represent the primary recognition element driving TaRGF5–TaRGI3 binding specificity. This finding aligns with experimental studies in Arabidopsis, where mutation of either the sulfotyrosine residue in RGF peptides or the corresponding arginine in RGI receptors abolishes signaling [48,59]. Collectively, these simulation results validate the docking-predicted interaction and establish that the TaRGF5–TaRGI3 interaction maintains structural integrity under physiologically relevant dynamic conditions. The stable, modification-dependent binding mode strongly suggests that wheat has retained the core molecular recognition mechanism operating in Arabidopsis, supporting the hypothesis that TaRGF5 functions as a bona fide ligand for TaRGI3 receptors in root development. Future experimental validation using site-directed mutagenesis of TyrS2 or Arg205, combined with in vivo complementation assays, would definitively test the functional requirement of this electrostatic anchor for TaRGF5 signaling in wheat.

A limitation of the molecular dynamics analysis is that each complex was evaluated using a single 100 ns trajectory rather than multiple independent simulations. In addition, several biological factors that may influence TaRGF5–TaRGI3 recognition in vivo were not incorporated into the computational models. The analysis suggests appropriate processing and post-translational modification of the TaRGF5 precursor, including tyrosine sulfation and proline hydroxylation, although the extent of these modifications in wheat remains to be experimentally established. The simulated environment also does not fully reproduce the physicochemical conditions of the wheat apoplast, where variations in pH and ionic strength could influence electrostatic interactions. Furthermore, predicted N-linked glycans on TaRGI3 were not included in the structural models and could potentially affect the accessibility or conformation of the ligand-binding region. Importantly, receptor–ligand recognition by LRR receptor kinases generally involves additional components of the signaling machinery, and the potential co-receptor(s) associated with TaRGI3 in wheat remain unknown. Collectively, these considerations indicate that the predicted TaRGF5–TaRGI3 interaction should be regarded as a computationally supported hypothesis, and experimental validation under physiologically relevant conditions is required.

## 4. Materials and Methods

### 4.1. Comprehensive Identification, Structural Features, Physicochemical Properties, Motif Analysis, Chromosomal Assignment, and Nomenclature of TaRGFs

The most recent assembly and annotation of the *T. aestivum* genome, released by the IWGSC, were used in this study to identify members of the RGF gene family. Specifically, genome data were retrieved from the RefSeq assembly version 2.1 (https://urgi.versailles.inrae.fr/download/iwgsc/IWGSC_RefSeq_Assemblies/v2.1/, accessed on 1 January 2026) along with its corresponding gene annotation version 2.1 (https://urgi.versailles.inrae.fr/download/iwgsc/IWGSC_RefSeq_Annotations/v2.1/, accessed on 1 January 2026). To ensure comprehensive identification of wheat *RGFs*, all known *A. thaliana* RGF protein sequences were retrieved from the TAIR database (http://www.arabidopsis.org/, accessed on 1 January 2026). These reference sequences were subsequently used as queries to perform local BLASTP searches against the wheat protein database with an E-value cutoff of 0.05 [60]. To recover additional divergent homologs, we then performed another BLASTP round in which the retained wheat sequences from the first search served as bait to identify further homologs. Given that full-length sequences may overlook divergent family members, we also conducted a supplementary BLASTP round using the conserved C-terminal functional domain to maximize the retrieval of all TaRGF candidates. Basic physicochemical properties of the identified TaRGFs, including amino acid length, molecular weight, and theoretical isoelectric point (pI), were computed using the ProtParam tool on the ExPASy server (https://web.expasy.org/protparam/, accessed on 1 January 2026; [61]). To predict the canonical signal peptide, the complete protein sequences of RGFs were submitted to SignalP 6.0 (https://services.healthtech.dtu.dk/services/SignalP-6.0/, accessed on 1 January 2026). The subcellular localization of TaRGFs was predicted using the DeepLoc server (https://services.healthtech.dtu.dk/services/DeepLoc-2.1/, accessed on 1 January 2026; [62]). Further, the Multiple Expectation Maximization for Motif Elicitation (MEME) Suite version 5.5.9 (https://meme-suite.org/meme/, accessed on 1 January 2026; [63]) was employed to identify up to five conserved motifs among the wheat RGFs. The potential biological functions of these motifs were subsequently annotated using the ScanProsite tool (https://prosite.expasy.org/scanprosite/, accessed on 1 January 2026; [64]).

For chromosomal assignment analysis, the localization of each *TaRGF* was retrieved from the wheat Unité de Recherche Génomique-Info (URGI) database and visualized using MapChart software (https://mybiosoftware.com/mapchart-2-2-graphical-presentation-linkage-maps-qtls.html, accessed on 1 January 2026). The genes were systematically designated according to their physical order along the chromosomes, following the nomenclature guidelines recommended by the wheat gene catalog (https://graingenes.org/GG3/wgc, accessed on 1 January 2026), ensuring consistent representation of homeologous genes across the A, B, and D subgenomes.

### 4.2. Sequence Alignment and Phylogenetic Analysis

RGF protein sequences from *A. thaliana* and *T. aestivum* were used for phylogenetic analysis. Mature protein sequences were analyzed as the full-length precursors following signal-peptide cleavage. Multiple sequence alignment was carried out using the CLUSTALW algorithm implemented in MEGA v11, with a 90% conservation cut-off applied to ensure alignment reliability [65]. Initial phylogenetic trees for the heuristic search were automatically generated using the neighbor-joining and bioNJ algorithms, based on a pairwise distance matrix computed with the Jones–Taylor–Thornton (JTT) substitution model. The topology displaying the highest log-likelihood value was retained for further analysis. Final phylogenetic inference was conducted using the maximum-likelihood method with the JTT matrix-based model [66]. The robustness of branching patterns was assessed by bootstrap analysis with 1000 replicates. The final tree was drawn to scale, with branch lengths representing the number of substitutions per site. Positions containing alignment gaps and missing data were excluded from the analysis to improve accuracy and confidence in evolutionary relationships.

### 4.3. RNA-Seq Analysis

#### 4.3.1. Dataset Collection

Publicly available RNA-seq datasets were retrieved from the NCBI Sequence Read Archive (SRA), the primary repository for high-throughput sequencing data enabling comparative transcriptomic analyses. Three wheat BioProjects were strategically selected to comprehensively investigate TaRGF expression dynamics across multiple developmental stages, including root and leaf tissues at 14, 20, and 25 days as well as stem tissue at 50 days. In addition, libraries from root tissues under drought stress were also selected to assess their expression pattern. These datasets encompassed: (1) PRJDB38512 (*T. aestivum* RNA-seq libraries sequenced on Illumina HiSeq 4000 (Illumina, San Diego, CA, USA); https://www.ncbi.nlm.nih.gov/Traces/study/?acc=DRP015447&o=sample_name_s%3Ad%3Bacc_s%3Aa&s=DRR813064, accessed on 1 April 2026); (2) PRJNA925925 (wheat cultivar Chuanmai104 RNA-seq sequenced using Illumina NovaSeq 6000 (Illumina, San Diego, CA, USA); https://www.ncbi.nlm.nih.gov/Traces/study/?acc=SRP418503&o=acc_s%3Aa, accessed on 1 April 2026); and (3) PRJNA369686 (wheat cultivar Chinese spring RNA-seq sequenced using Illumina HiSeq 2000 (Illumina, San Diego, CA, USA); https://www.ncbi.nlm.nih.gov/Traces/study/?acc=SRP098756&o=acc_s%3Aa, accessed on 1 April 2026). All datasets consisted of paired-end Illumina reads (Table S3).

#### 4.3.2. Quality Control Pipeline

Comprehensive quality assessment of raw FASTQ files was performed using FastQC (v0.11.9) to evaluate sequence quality scores, adapter contamination, overrepresented sequences, and GC bias patterns [67]. Subsequent read trimming and filtering employed Trimmomatic (v0.39) with stringent parameters, including adapter removal, quality-based trimming, endpoint quality filtering, and minimum length retention [68]. This rigorous quality control pipeline ensured only high-confidence, contaminant-free reads proceeded to alignment, which is essential for accurate quantification of TaRGF expression levels.

#### 4.3.3. Read Mapping and Transcript Quantification

High-quality filtered reads were aligned to a custom reference comprising 24 TaRGFs full-length cDNA sequences using Bowtie2 (v2.4.5) in end-to-end sensitive local alignment mode, optimized for transcriptomic mapping [69]. Uniquely mapped reads were retained for homeolog-specific quantification, whereas reads mapping equally well to multiple homeologs were excluded. Transcript assembly and abundance estimation were performed with StringTie (v2.2.1) using the reference annotation (-G), reference-guided quantification (-e), and conservative thresholds of -f 0.05, -c 1.5, -s 4.75, and -j 2 to reduce unsupported splice-variant predictions [70]. Transcript assembly and abundance estimation were conducted with StringTie, which quantified gene expression as transcripts per million (TPM) values. TPM normalization accounts for both sequencing depth and gene length variation, providing robust, comparable expression metrics across biological replicates, genotypes, and experimental conditions.

#### 4.3.4. Statistical Analysis and Visualization

All statistical analyses and visualizations were executed in the R programming environment (v4.3.0, https://www.r-project.org/, accessed on 1 April 2026; [71]). TaRGF reference transcript abundance was represented using TPM values and transformed as E_i_ = log_2_(TPM_i_). Reference transcripts with a TPM value of zero were assigned a minimum value of 1.0 × 10^−15^ before transformation, producing a lower plotting limit of approximately −49.83. Heatmap generation and hierarchical clustering utilized the pheatmap package (v1.0.12), featuring log_2_ normalization across genes and samples, complete linkage clustering with Euclidean distance metrics, and color gradient representation of expression levels.

### 4.4. Plant Materials and Drought Treatment

A drought-susceptible wheat cultivar, Sids 13, was used in the drought experiment. Plants were grown in 15 cm diameter pots containing a 1:1:1 (*v*/*v*) mixture of washed sand, peat moss, and clay under controlled growth-chamber conditions at 25 °C with a 16 h photoperiod and regular irrigation. The drought treatment was initiated when the seedlings were 7 days old. This time point was designated as 0 days of drought treatment. For the drought treatment, water was withheld from the designated plants, whereas control plants were maintained under the same environmental conditions and watered regularly. Root samples were collected at four time points: 7-day-old seedlings at 0 days of drought treatment, 14-day-old plants after 7 days of drought treatment, 20-day-old plants after 14 days of drought treatment, and 25-day-old plants after 21 days of drought treatment. The collected root tissues were immediately frozen in liquid nitrogen and stored at −80 °C until RNA extraction.

### 4.5. RNA Isolation, Primer Design, and qRT-PCR Analysis

Total RNA was extracted from harvested root tissues using TRIzol reagent (Invitrogen, Carlsbad, CA, USA) according to the manufacturer’s protocol. The isolated RNA was subsequently treated with the Ambion TURBO RNase-Free DNase kit (Thermo Fisher Scientific, Waltham, MA, USA) following the manufacturer’s instructions to remove residual genomic DNA contamination. Complementary DNA was subsequently synthesized from 1 μg of total RNA using Revert Aid Premium Reverse Transcriptase (Thermo Scientific™, USA). The three homeologs of wheat RGF5 (TaRGF5A, TaRGF5B, and TaRGF5D) were selected for expression analysis based on their expression that was identified in prior transcriptomic profiling. Normalization of target gene transcript levels was achieved using the reference gene translation elongation factor 1α (EF1α). Quantitative real-time PCR (qRT-PCR) was performed using the primer pairs and their expected molecular sizes listed in Table S4, with each 20 µL reaction containing 1 µL cDNA, 12.5 µL 2× iTaq SYBR Green Supermix (Bio-Rad, Tokyo, Janpan), 0.75 µL of 1:100 diluted ROX reference dye, 1 µL of each 10 µM forward and reverse primer, and nuclease-free water to bring the total volume to 20 µL. All reactions were conducted in triplicate. Amplification was performed using an Mx3005P qPCR System (Stratagene, La Jolla, CA, USA) under the following cycling conditions: initial denaturation at 95 °C for 5 min, followed by 40 cycles of 95 °C for 30 s, 60 °C for 60 s, and 72 °C for 20 s, with a final extension at 72 °C for 10 min. Relative expression levels of the TaRGF5 homeologs were calculated using MxPro QPCR Software v4.10 based on cycle threshold (Ct) values, with EF1α used as the reference gene for normalization. The normalized expression values represent relative mRNA abundance in each sample.

### 4.6. Promoter Prediction and Cis-Regulatory Element Analysis

For promoter prediction of *TaRGFs*, a 2000 bp genomic region upstream of each predicted TSS was extracted and considered a putative promoter sequence. Initial promoter region screening was carried out using the BDGP promoter prediction tool (https://www.fruitfly.org/seq_tools/splice.html; accessed on 1 March 2026, [72]), applying a score cutoff threshold of 0.80 to screen all potential transcription start sites (TSSs). To enhance prediction reliability, the sequences were further analyzed using the Promoter 2.0 Prediction Server (https://services.healthtech.dtu.dk/services/Promoter-2.0/, accessed on 1 March 2026; [73]). These tools were used to screen the promoter regions for the maximum number of predicted regulatory elements.

Subsequent identification of cis-regulatory elements within the predicted promoter regions was performed using the PLACE (Plant Cis-Acting Regulatory DNA Elements) database [74]. Additionally, transcription factor binding site (TFBS) prediction was conducted through PlantRegMap, providing a detailed overview of potential transcriptional regulatory motifs (https://plantregmap.gao-lab.org/funtfbs_manual.php, accessed on 1 March 2026; [75]). The resulting TFBS dataset was exported to Microsoft Excel for further processing. Entries with a prediction score greater than 10 were retained to ensure high-confidence binding site identification, and the retained sites were sorted according to their genomic start positions in ascending order. Finally, the filtered regulatory elements were visualized exclusively in graphical form using the R program, version 4.3.0 (https://www.r-project.org/, accessed on 1 March 2026; [66]), displaying the positional distribution of predicted cis-elements and transcription factor binding sites along each promoter region.

### 4.7. Molecular Docking Analysis

#### 4.7.1. Ligand Preparation and Post-Translational Modification

To prepare the peptide ligands, we first retrieved the C-terminal sequences encoding the mature peptide from each of the three wheat subgenomes (A, B, and D). De novo three-dimensional structures of the three wheat homeologs of the peptide were predicted using the PEP-FOLD 3.5 server, which is optimized for short peptide modeling based on structure and fragment assembly [76]. To approximate the biologically active form of these peptides, post-translational modifications (PTMs) were introduced using the CHARMM-GUI PDB Reader and Manipulator [77].

#### 4.7.2. Putative RGF Receptor Identification and Homology Modeling

Wheat RGF5 candidate interactor homeologs were identified through a BLASTP search of the wheat proteome (IWGSC RefSeq v2.1) using AtRGI3 as a query. The top three hits mapped to the A, B, and D subgenomes of TaRGI3 receptors were selected. Three-dimensional structural models of the candidate wheat receptor proteins were generated using AlphaFold2, a deep learning-based approach that enables highly accurate protein structure prediction from amino acid sequences [78]. For subsequent molecular docking analyses, only the extracellular leucine-rich repeat (LRR) ectodomain was retained, corresponding to residues 24–700. To improve structural reliability, low-confidence regions were excluded from the models by removing residues with predicted local distance difference test (pLDDT) scores below 50. Such regions are generally associated with intrinsically disordered segments or poorly resolved structural predictions in AlphaFold2 models [78].

#### 4.7.3. Peptide-Protein Docking Analysis

Molecular docking analysis was carried out using the HADDOCK 2.4 web server, which guides docking through ambiguous interaction restraints (AIRs) derived from experimental or predicted interaction data (https://wenmr.science.uu.nl/haddock2.4/, accessed on 1 March 2026). A set of active residues was used to generate AIRs that bias the docking toward biologically relevant interaction regions. For TaRGI3, active residues were defined as 205, 207, 208, 228, 230, 232, 254, and 256, based on predicted surface accessibility and their proximity to the conserved sulfotyrosine-binding region. For TaRGF5, the entire 13-residue mature peptide was defined as active, given its small size and the established role of the full sequence in receptor engagement (Table S5). Passive residues were assigned automatically by HADDOCK. A docking simulation was performed for the 13-amino-acid peptide incorporating key PTMs, including sulfotyrosine at position 2 (Tyr2S) and hydroxyproline at position 10 (HyPro10), which are critical for RGF activity. These modified residues were built directly into the peptide structure prior to submission, so the docking run itself was carried out on the fully modified peptide rather than a naive sequence with PTMs added afterward. As a reference control, the experimentally validated Arabidopsis complex was reproduced by docking the 13-amino-acid AtRGF5 peptide (with PTMs) to the AtRGI3 LRR ectodomain (PDB ID: 5HZ3; https://www.rcsb.org/structure/5HZ3, accessed on 1 May 2026). This structure has been resolved crystallographically and serves as a benchmark for RGF–RGI interactions, enabling comparative evaluation of the wheat docking analyses. Binding interfaces were carefully mapped to document key intermolecular contacts, including hydrogen bonds, salt bridges, hydrophobic contacts, and pi–cation interactions, between receptor and peptide residues. All docking runs used the HADDOCK 2.4 easy-interface default settings, without modification: 1000 rigid-body models were generated in the initial stage, and the best 200 were carried forward through semiflexible refinement and then explicit solvent refinement. Final models were clustered using HADDOCK’s default fraction of common contacts (FCC) method, with a cutoff of 0.60 and a minimum cluster size of 4. The wheat model was selected from Cluster 2 (24 members) and the Arabidopsis reference model from Cluster 1 (200 members), each representing the lowest HADDOCK score within its respective cluster.

#### 4.7.4. Interaction Analysis and Visualization

To characterize the molecular determinants of receptor–peptide recognition, a multi-step interaction analysis and visualization workflow was performed. The top-scoring clusters from each HADDOCK run were selected based on interface RMSD (iRMSD < 2.0 Å). Energy decomposition was then performed on each selected complex to extract van der Waals, electrostatic, and desolvation energy terms, providing a detailed profile of the forces driving each receptor–peptide interaction. All complexes were visualized using PyMOL v2.5 and Discovery Studio Visualizer (https://www.pymol.org/, accessed on 1 May 2026). Binding interfaces were carefully mapped to document key intermolecular contacts, including hydrogen bonds, salt bridges, hydrophobic contacts, and pi–cation interactions, between receptor residues and peptide residues.

### 4.8. Molecular Dynamics Simulation Analyses

#### 4.8.1. System Preparation and Parameterization

All simulations were performed using the AMBER ff14SB force field to describe the protein receptor, given its reliability for structured proteins and protein–ligand interactions [79]. The solvent environment was modeled using the TIP3P explicit water model, which provides a strong balance between computational efficiency and accuracy [80].

The TaRGF5 peptide involved two key post-translational modifications, sulfotyrosine (TyrS2) and hydroxyproline (HyPro10), which were not fully supported in standard AMBER libraries. Sulfotyrosine was parameterized using a custom approach, combining ff14SB-compatible aromatic parameters with a locally generated frcmod file and mol2-based atom typing to ensure appropriate bonded terms and AMBER-compatible partial charges for the sulfate group [81]. Hydroxyproline was described using available AMBER-compatible parameters. System setup was performed using the tleap module in AmberTools (v20.0) [82]. Hydrogen atoms were added, and disulfide bonds between cysteine residues within a 2.5 Å distance threshold were automatically assigned (CYX residues). The complex was solvated in a cubic TIP3P water box with a minimum buffer distance of 12.0 Å from the protein surface to the box edges to prevent periodic artifacts. To neutralize the system and provide a minimal salt concentration, 15 Na^+^ and 15 Cl^−^ ions were randomly added, resulting in a solvated system with final periodic box dimensions of approximately 168.6 × 103.5 × 92.5 Å.

#### 4.8.2. Molecular Dynamics Simulation Protocol

All molecular dynamics simulations were performed using OpenMM (version 7.5) with GPU acceleration [83]. Long-range electrostatics were treated using the Particle Mesh Ewald (PME) method with a non-bonded cutoff of 1.0–1.2 nm, and all bonds involving hydrogen atoms were constrained using the SHAKE algorithm, enabling a 2.0 fs time step. The simulation protocol included 5000 steps of energy minimization, followed by 3 nanoseconds (ns) of equilibration under the NPT ensemble at 300 K using a Langevin integrator (friction coefficient 1.0 ps^−1^) and 1 bar using a Monte Carlo barostat (volume updates every 25 steps) [84]. A 100 ns production run was then conducted under the same conditions, with coordinates and energy data recorded every 10 ps for analysis.

#### 4.8.3. Trajectory Analysis

Trajectory analyses, including root mean square deviation (RMSD), root mean square fluctuation (RMSF), intermolecular hydrogen bonding, and electrostatic interaction assessment, were performed using VMD (version 1.9.3) with built-in tools and custom Tcl scripts. Structural visualizations were also generated in VMD [85]. Graphical representations and statistical analyses were conducted using Matplotlib, v3.10.9 [86].

## 5. Conclusions

This study establishes the first comprehensive genomic and computational framework for understanding RGF-mediated root development in wheat. The identification of 15 TaRGF genes, their phylogenetic relationships, tissue-specific expression patterns, and predicted receptor interactions provides a valuable resource for the wheat research community. The root-enriched expression of TaRGF5, its repression under drought stress, and the computational validation of its interaction with TaRGI3 receptors collectively suggest this peptide–receptor module as a candidate regulator of root growth plasticity under water-limited conditions. TaRGF5 and TaRGI3 represent putative targets for genetic improvement of wheat root systems. CRISPR-Cas9-mediated knockout or promoter editing could be used to modulate TaRGF5 expression levels, potentially uncoupling root growth from drought-induced repression and enabling sustained root development under water stress. As climate change intensifies drought frequency and severity in major wheat-growing regions, understanding and harnessing the TaRGF5–TaRGI3 signaling module may contribute to developing more resilient wheat varieties with root systems optimized for water-limited environments.

## Figures and Tables

**Table 1:** Comparison of docking scores and energy components for the AtRGF5–AtRGI3 and TaRGF5–TaRGI3 interactions.

Score	AtRGF5–AtRGI3	TaRGF5–TaRGI3
HADDOCK score	−202.4 ± 1.2	−105.3 ± 2.8
Van der Waals energy	−71.7 ± 5.3	−27.8 ± 2.5
Electrostatic energy	−624.8 ± 42.2	−397.7 ± 18.0
Desolvation energy	−8.5 ± 2.1	−2.5 ± 1.0

## Data Availability

The original contributions presented in this study are included in the article/Supplementary Material. Further inquiries can be directed to the corresponding author.

## Supplementary Materials

The following supporting information can be downloaded at https://www.mdpi.com/article/10.3390/ijms27177616/s1.
